# Developmental emergence of cortical neurogliaform cell diversity

**DOI:** 10.1242/dev.201830

**Published:** 2023-08-01

**Authors:** Lucia Gomez, Christelle Cadilhac, Julien Prados, Nandkishor Mule, Denis Jabaudon, Alexandre Dayer

**Affiliations:** ^1^ Department of Basic Neurosciences, University of Geneva, 1211 Geneva, Switzerland; ^2^ Clinic of Neurology, Geneva University Hospital, 1211 Geneva, Switzerland; ^3^ Department of Psychiatry, Geneva University Hospital, 1205 Geneva, Switzerland

**Keywords:** Neurogliaform, Interneuron, Cortex, Diversity, Development, Transcriptomics, Mouse

## Abstract

GABAergic interneurons are key regulators of cortical circuit function. Among the dozens of reported transcriptionally distinct subtypes of cortical interneurons, neurogliaform cells (NGCs) are unique: they are recruited by long-range excitatory inputs, are a source of slow cortical inhibition and are able to modulate the activity of large neuronal populations. Despite their functional relevance, the developmental emergence and diversity of NGCs remains unclear. Here, by combining single-cell transcriptomics, genetic fate mapping, and electrophysiological and morphological characterization, we reveal that discrete molecular subtypes of NGCs, with distinctive anatomical and molecular profiles, populate the mouse neocortex. Furthermore, we show that NGC subtypes emerge gradually through development, as incipient discriminant molecular signatures are apparent in preoptic area (POA)-born NGC precursors. By identifying NGC developmentally conserved transcriptional programs, we report that the transcription factor *Tox2* constitutes an identity hallmark across NGC subtypes. Using CRISPR-Cas9-mediated genetic loss of function, we show that *Tox2* is essential for NGC development: POA-born cells lacking *Tox2* fail to differentiate into NGCs. Together, these results reveal that NGCs are born from a spatially restricted pool of *Tox2^+^* POA precursors, after which intra-type diverging molecular programs are gradually acquired post-mitotically and result in functionally and molecularly discrete NGC cortical subtypes.

## Teaser

**Summary:** Genetic fate mapping, single-cell transcriptomics, and electrophysiological recordings reveal that mouse cortical neurogliaform interneurons with distinct functional and anatomical properties develop from a spatially restricted pool of *Tox2*-expressing precursors in the preoptic area.

## INTRODUCTION

Cortical interneurons (INs) regulate microcircuit excitability (Isaacson and Scanziani, 2011) and belong to distinct molecular types with characteristic morphologies, electrophysiological properties, connectivity patterns, laminar distributions and developmental origins (Gouwens et al., 2020; Jiang et al., 2015; Lim et al., 2018). Using single-cell transcriptomics, over 60 subtypes of cortical INs have been identified, organized into six main classes: *Pvalb*^+^, *Sst*^+^, *Vip*^+^, *Serpinf1*^+^, *Sncg*^+^ and *Lamp5*^+^ (Tasic et al., 2018). Although molecular taxonomies have shed light on inhibitory neuron diversity in the mature cortex, when and where each of these distinct IN populations are generated and how subtype diversity arises during development remains unclear. In particular, whereas the developmental trajectories of medial ganglionic eminence (MGE)-born INs are increasingly understood (Allaway et al., 2021; Lim et al., 2018; Llorca and Deogracias, 2022), INs generated in other embryonic regions remain less explored and questions on their differentiation and diversification are still partially answered. In particular, the developmental emergence and maturation of neurogliaform cells (NGCs) originating from a spatially restricted domain in the preoptic area (POA) and expressing *Hmx3* post-mitotically (Niquille et al., 2018) remain undescribed.

NGCs are members of the *Htr3a*-expressing family of INs. They predominantly locate in upper cortical layers (ULs) and display extensive dense axonal arborizations able to modulate the activity of large neuronal populations, even crossing cortical areas (Jiang et al., 2015; Sakalar et al., 2022; Tremblay et al., 2016). NGCs are thought to be central for bottom-up and top-down integration of cortical signals, as they receive long-range corticocortical, neuromodulatory subcortical and thalamocortical inputs (Colonnese et al., 2021; Ibrahim et al., 2021). Moreover, NGCs are a significant source of ‘slow’ cortical inhibition through GABA_B_-mediated volumetric transmission (Ozsvár et al., 2021; Sanchez-Vives et al., 2021) and may thereby regulate gain modulation and coincidence detection during cortical associative tasks (Cohen-Kashi Malina et al., 2021; Hou and Capogna, 2018; Poorthuis et al., 2018).

Here, we investigated cortical NGC developmental emergence by combining intersectional genetic fate mapping, single-cell transcriptomics, and electrophysiological recordings. Specifically, using *Hmx3*-Cre::*Htr3a*-GFP;Ai14 (*Rosa26R*-tdTOM^fl/fl^) transgenic mice to label previously described NGCs (Niquille et al., 2018), we reveal that different molecular subtypes of NGCs exhibiting distinct functional and anatomical properties exist in the neocortex. Following their birth in the embryonic POA, we observed that NGC gene expression programs diverge early post-mitotically to generate two main subtypes of cells: Dock5^+^NGCs and Lsp1^+^NGCs, which depend on the transcription factor (TF) *Tox2* for their maturation.

Together, our results reveal an unexpected level of functional, molecular and anatomical diversity within NGCs that is gradually acquired post-mitotically and emerges from a *Tox2*-expressing, spatially restricted pool of POA progenitors.

## RESULTS

**Fig. 1.**
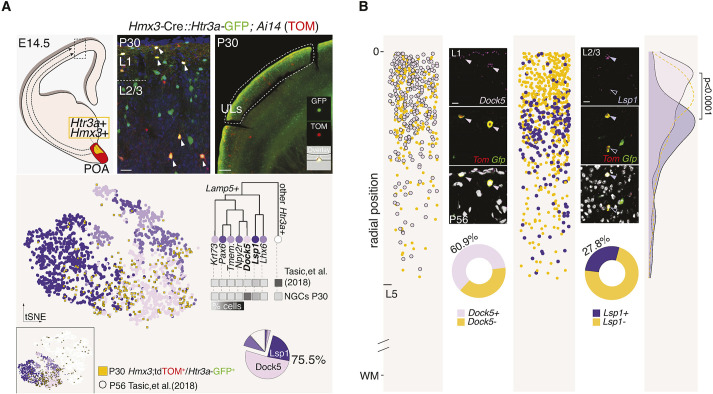
**UL NGCs consist of two transcriptionally defined subtypes.** (A) Schematic illustrating the procedure for microdissection and single-cell capture for RNA-seq of UL cortical INs from *Hmx3*-Cre::*Htr3a*-GFP;Ai14 (*Rosa26R*-tdTOM^fl/fl^) postnatal mice. Schematic of an E14.5 coronal section through the preoptic area (POA) where the red shaded region represents the POA region populated by Hmx3-expressing cells and the yellow shaded region represents the POA micro-domain populated by cells expressing both Htr3a and Hmx3. Dashed arrows illustrate the migration process of POA-derived Htr3a^+^Hmx3^+^ cells towards the neocortex (dashed rectangular box). Left image shows a P30 coronal section through the cortex containing post-migratory POA-derived Htr3a^+^Hmx3^+^ interneurons (arrowheads). Right image illustrates the upper layers (ULs) of a cortical region micro-dissected for RNA sequencing (dashed region) (insets show one of these cells captured in a micro-fluidic chamber for sequencing). tSNE plot shows the integration coordinates of sequenced P30 NGCs that passed quality control (*Hmx3*;tdTOM^+^/*Htr3a*-GFP^+^ cells, *n*=301) onto P56 cortical *Htr3a*-expressing INs (*n*=4743) (Tasic et al., 2018) (inset tSNE covers all *Htr3a*-expressing IN subtypes, main tSNE displays only *Htr3a*^+^*Lamp5*^+^ INs). Hierarchical tree (adapted from Tasic et al., 2018) illustrating the common root and transcriptomic proximity of *Lamp5*-expressing subtypes and the splitting point on the parent node of *Htr3a*-expressing INs obtained from the Tasic et al. (2018) dataset (*Lamp5*-expressing subtypes: *Krt73*^+^, *Pax6*^+^, *Tmem182*^+^, *Npy2r*^+^, *Dock5*^+^, *Lsp1*^+^ and *Lhx6*^+^, see subtype definitions below). Heatmap shows the percentage subtype enrichment of P30 NGCs (*Hmx3*;tdTOM^+^/*Htr3a*-GFP^+^) versus non-NGC enriched pan-INs sampling (Tasic et al., 2018) (raw data available in Table S1 and detailed frequencies in Table S2) with darker shades representing a higher percentage of cells. Pie chart displays the percentage of P30 NGCs mapping to each *Htr3a*-expressing IN subtype [highlighted NGCs fraction mapping to Dock5^+^ NGC and Lsp1^+^ NGC subtypes, 51.7% (*n*=76) and 23.8% (*n*=35), respectively; total 75.5%: 111 out of 147 assigned cells, remaining 154 cells failed to reach a consensus prediction] (raw data in Table S1 and detailed frequencies in Table S2). (B) smFISH validation of *Dock5* and *Lsp1* expression in P56 *Hmx3*;tdTOM^+^/*Htr3a*-GFP^+^ cells (NGCs) (479 cells for *Dock5* quantification, 5 technical replicates; 746 cells for *Lsp1* quantification, 5 technical replicates, independent experiments). Images illustrate the mRNA expression of these markers in fate-mapped NGCs (filled arrowheads indicate Dock5^+^ or Lsp1^+^ NGCs and unfilled arrowheads indicate Lsp1^−^ NGCs). Pie charts represent the mean percentage of NGCs expressing either *Dock5* or *Lsp1* mRNAs across ULs [60.9% (*n*=292, s.d.=23) and 27.8% (*n*=208, s.d.=9), respectively]. Scatter plots detail the cortical position of quantified NGCs across cortical ULs depth (distance relative to pia surface – set as value 0) color-coded by their expression of *Dock5*, *Lsp1* or neither (only *tdTom* and *Gfp*) (single-cell categorization and position as well as detailed frequencies available in Table S3). Density plot displays the laminar distribution of quantified NGCs according to their gene expression profiles (D=0.19, *P*-value=4.45×10^−9^; Kolmogorov–Smirnov two-sided test). Color-coding: Dock5^+^NGCs or *Dock5* mRNA expression (pink), Lsp1^−^NGCs or *Lsp1* mRNA expression (purple), all NGCs (yellow), *Lamp5*^+^ subtypes (shades of purple), *Lamp5*^−^ subtypes (white). Color-shape-coding: *Hmx3*;tdTOM^+^/*Htr3a*-GFP^+^ cells (yellow squares or circles), *Lamp5*^+^ specific subtypes (shades of purple circles), *Lamp5*- subtypes (white). Subtype definitions: *Krt73* (Tasic et al., 2018 subtype *Lamp5 Krt73*), *Pax6* (*Lamp5 Fam19a1 Pax6*), *Tmem*. (*Lamp5 Fam19a1 Tmem182*), *Npy2r* (*Lamp5 Ntn1 Npy2r*), *Dock5* (*Lamp5 Plch2 Dock5*), *Lsp1* (*Lamp5 Lsp1*) and *Lhx6* (*Lamp5 Lhx6*). L, layer; ULs, upper layers; WM, white matter. Scale bars: 25 µm (A, high magnification); 500 µm (A, low magnification); 10 µm (B).

NGCs, a subgroup of *Htr3a*-expressing INs, are specifically labeled in *Hmx3*-Cre::*Htr3a*-GFP;Ai14 mice (Niquille et al., 2018). In order to investigate the emergence of their molecular diversity, we collected *Hmx3*;tdTOM^+^/*Htr3a*-GFP^+^ (POA-born) (Fig. 1A, Figs S1, S2A) and *Hmx3*;tdTOM^−^/*Htr3a*-GFP^+^ [caudal ganglionic eminence (CGE)-born] UL INs (Fig. S3A,B) at postnatal days (P) 15 and P30 using fluorescence-activated cell sorting (FACS), and performed single-cell RNA sequencing (scRNA-seq).

### UL NGCs consist of two transcriptionally defined subtypes

To determine the transcriptional subtype(s) to which POA-born cortical NGCs belong, we mapped and label-transferred UL-sorted and sequenced NGCs onto adult *Htr3a*-expressing INs (Tasic et al., 2018) (Fig. 1A, Figs S2A, S3, Table S1). Machine learning approaches (see Materials and Methods; Figs S2B, S3A,B) revealed that most of collected POA-born INs (75.5% at P30 and 62.2% at P15) belong to two previously identified *Htr3a*^+^ transcriptional subtypes: *Lamp5*^+^ *Plch2*^+^ *Dock5*^+^ cells (hereafter called Dock5^+^NGCs; 51.7% of all *Htr3a*^+^ cells at P30 and 32.4% at P15; 68.4% of NGCs at P30 and 52.2% at P15) and *Lamp5*^+^ *Lsp1*^+^ cells (hereafter called Lsp1^+^NGCs; 23.8% of all *Htr3a*^+^ cells at P30 and 29.7% at P15; 31.5% of NGCs at P30 and 47.8% at P15) (Fig. 1A, Fig. S2A, Table S2). CGE-born INs (i.e. *Hmx3*;tdTOM^−^/*Htr3a*-GFP^+^) mapped cell types instead distributed in a complementary manner (77.4% Dock5^−^Lsp1^−^ at P30; 78.8% Dock5^−^Lsp1^−^ at P15) (Fig. S3A,B, Table S2). Of the *Lamp5*^+^*Dock5*^+^ and *Lamp5*^+^*Lsp1*^+^ cells assigned in both mouse lines, the vast majority were labeled by the *Hmx3*-Cre;tdTOM approach, suggesting that it provides high efficiency labeling for these subtypes [at P30: 79.8% (100% *Lamp5*^+^Dock5^+^ and 55.5% *Lamp5*^+^*Lsp1*^+^); at P15: 74.2% (90% *Lamp5*^+^*Dock5*^+^ and 62.3% *Lamp5*^+^*Lsp1*^+^)]. These results suggest that, within the diverse *Htr3a*-expressing IN family composed of 29 subtypes (Tasic et al., 2018), both Dock5^+^NGCs and Lsp1^+^NGCs belong to the *Hmx3* lineage (Fig. 1A, Fig. S2A) and that genetic fate mapping is a useful technique to enhance the capture of NGC subtypes (Fig. 1A, Table S2).

We next assessed whether these two main molecular subtypes of NGCs show different laminar distributions in the cortex. For this purpose, we used single-molecule fluorescence *in situ* hybridization (smFISH) to detect the expression of *Dock5* or *Lsp1* transcripts in ULs fate-mapped NGCs at P56 (Fig. 1B, Table S3). smFISH quantification results supported the NGC-subtype proportions found in ULs using scRNA-seq (60.9% *Dock5*^+^NGCs and 27.8% *Lsp1*^+^NGCs) and revealed specific laminar enrichments for NGC-subtypes: whereas *Dock5*^+^NGCs locate preferentially in L1 (70%; Table S3), *Lsp1*^+^NGCs distribute more evenly across ULs, peaking in L2/3 (Fig. 1B) and are also occasionally observed in cortical deep layers (DLs).

Together, these results reveal that POA-born NGCs located in cortical ULs are diverse, belonging to two main *Lamp5*^+^ molecular subtypes. Moreover, *Dock5*^+^NGCs and *Lsp1*^+^NGCs display complementary laminar distributions.

### NGC type and subtype molecular signatures

**Fig. 2.**
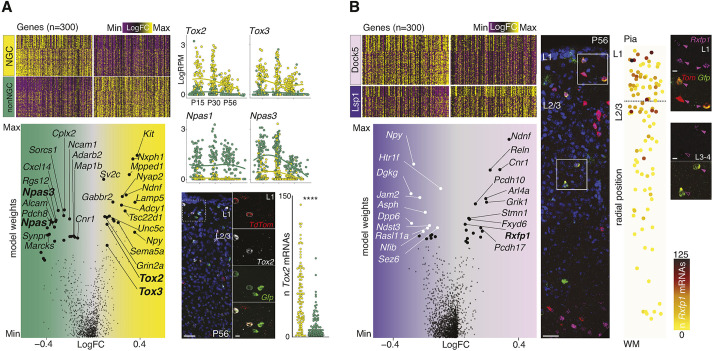
**NGC type and subtype molecular architectures.** (A) Heatmap displaying gene expression fold change (FC) values for NGCs (yellow) versus non-NGC (other *Htr3a*^+^ INs) (green) postnatally conserved DEGs (columns) across maturation-ordered cells (rows) (see detailed FC and SVM weight per gene in the model, *n*=300, 150 per type, in Table S5). Color gradient: magenta, low; black, mid; yellow, high. Volcano plot representing genes ranked by FC and SVM weights (names for the top 15 genes per IN type are highlighted, with transcription factor (TF) family groups per type in bold). Scatter plots with LOESS fitting illustrate gene expression from P15 to P56 for bold-highlighted TFs on NGCs (yellow) and non-NGCs (green). Low-magnification and high-magnification (inset) images representing the smFISH experiment for *Tox2*, *Gfp* and *Tom* at P56 in *Hmx3*-Cre::*Htr3a*-GFP;Ai14 mouse cortical sections. Bottom scatter plot represents the single-cell quantitative analysis of *Tox2* mRNAs for NGCs (yellow) and non-NGCs (green). *y*-axis represents the number of mRNA molecules detected per single cell (unpaired *t*-test, ****P<0.0001; *n*=3 brains, 282 cells) (Table S7). (B) Heatmap displaying gene expression FC values for Dock5^+^NGCs (pink) versus Lsp1^+^NGCs (purple) postnatally conserved DEGs (columns) across maturation-ordered cells (rows) (see detailed FC and SVM weight per gene in the model, *n*=300, 150 per type, in Table S6). Volcano plot representing genes ranked by FC and SVM weights (names for the top 15 genes per IN type are highlighted, with *Rxfp1*, a Dock5^+^NGC-specific gene validated *in situ* in bold). Low- and high-magnification (inset) images illustrating the smFISH experiment for *Rxfp1*, *Gfp* and *Tom* at P56 on *Hmx3*-Cre::*Htr3a*-GFP;Ai14 mouse cortical sections (filled arrowheads indicate Rxfp1^+^ cells and unfilled arrowheads indicate Rxfp1^−^ cells). Scatter plot representing single-cell quantitative assessment of *Rxfp1* expression across cortical depth (*y*-axis) (*n*=3 brains, 108 cells) (Table S8). Color gradient indicates the number of *Rxfp1* mRNA molecules detected on each single cell (yellow, low; brown, mid; black, high). Color-coding: FC gradient (magenta, low; black, mid; yellow, high); NGC type (yellow), non-NGC *Htr3a*^+^ INs (green), Dock5^+^NGC subtype (pink), Lsp1^+^NGC subtype (purple); *Rxfp1* mRNA smFISH count gradient (yellow, low; brown, mid; black, high). LogFC, Log1p fold change; L, layer, WM, white matter. Scale bars: 50 µm (A, low magnification); 10 µm (A, high magnification; B, high magnification); 25 µm (B, low magnification).

Next, we aimed at identifying the molecular signatures characterizing the NGC type (composed of Dock5^+^NGCs and Lsp1^+^NGCs), versus other *Htr3a*^+^ INs and each NGC subtype (Dock5^+^NGCs versus Lsp1^+^NGCs) (Fig. 2A,B, Table S4). To do so, we first compared postnatally conserved gene expression patterns in NGCs (belonging to *Dock5*^+^ and *Lsp1*^+^ subtypes) with the related non-NGC IN population (*Dock5*^−^ or *Lsp1*^−^ INs; i.e. *Htr3a*^+^ CGE-born INs) (Fig. 2A). Using support vector machine (SVM) classification (see Materials and Methods), we identified the top 150 transcripts distinguishing each population from P15 to adulthood and found that, whereas the TFs *Tox2* and *Tox3* are enriched in NGCs, *Npas1* and *Npas3* are enriched in CGE-born INs (Table S5). We validated *Tox2* differential expression in NGCs versus other *Htr3a*^+^ INs quantitatively using smFISH (Fig. 2A, Table S7). Although *Tox2* is a specific marker for NGCs compared with other members of the *Htr3a*-expressing IN family, lower RNA expression levels of *Tox2* are detectable in other cortical cell types (Fig. S4D,E). Gene enrichment analysis on the identified core gene sets (Fig. S4A-C, Table S5) further revealed that NGCs express more synaptic release-related transcripts compared with non-NGCs (e.g. *Syt1*, *Syt7*, *Rims1*), potentially reflecting specificities in vesicle exocytosis (Kaeser et al., 2012).

We then set out to find out the molecular signatures distinguishing NGC subtypes (Dock5^+^NGCs versus Lsp1^+^NGCs) throughout postnatal maturation (Fig. 2B, Table S6). We found that *Ndnf* and *Npy* were differentially expressed in Dock5^+^NGCs and Lsp1^+^NGCs, respectively (Fig. 2B). This finding indicates that Dock5^+^NGCs likely correspond to the previously named canopy cells (NDNF^+^ NPY-low NGCs), mostly located in L1 (Schuman et al., 2019), whereas Lsp1^+^NGCs would correspond to NDNF-low NPY^+^ NGCs, mostly located in L2-6. In addition, among the top NGC-subtype distinctive markers, we identified the relaxin family receptor 1 (*Rxfp1*) as a specific marker for Dock5^+^NGCs. Using smFISH, we confirmed that *Rxfp1* is expressed preferentially in UL NGCs at P56, where Dock5^+^NGCs predominate (Figs 1B, 2B, Tables S3, S8). Gene enrichment analysis contrasting the two NGC subtypes (Fig. S5, Table S6) indicated that Dock5^+^NGCs expressed abundant cell adhesion molecule-related transcripts compared with Lsp1^+^NGCs. Notably, the protocadherins *Pcdh10* and *Pcdh17* are enriched in Dock5^+^NGCs (Fig. S5), suggesting the existence of cell–cell interaction mechanisms that could mediate NGC subtype-specialized contacts (Lefebvre et al., 2012). In addition, we observed a differential enrichment of glutamate versus GABA receptor expression in Dock5^+^NGCs and Lsp1^+^NGCs, respectively (Fig. S5), likely reflecting distinct synaptic partners and inputs for each subtype. Specifically, whereas transcripts for the glutamate ionotropic receptors subunits *Grik1*, *Grik2* and *Grin3a* were more expressed in Dock5^+^NGCs, Lsp1^+^NGCs were enriched in genes encoding for GABA type-A receptor subunits such as *Gabrd* and *Gabra5*.

Altogether, these results suggest that molecularly defined NGC subtypes may play distinct roles in cortical circuits, as proposed by their differential expression of transcripts for neuropeptides, cell adhesion molecules, synaptic regulators and receptors.

### Functional and anatomical correlates of NGC subtypes

**Fig. 3.**
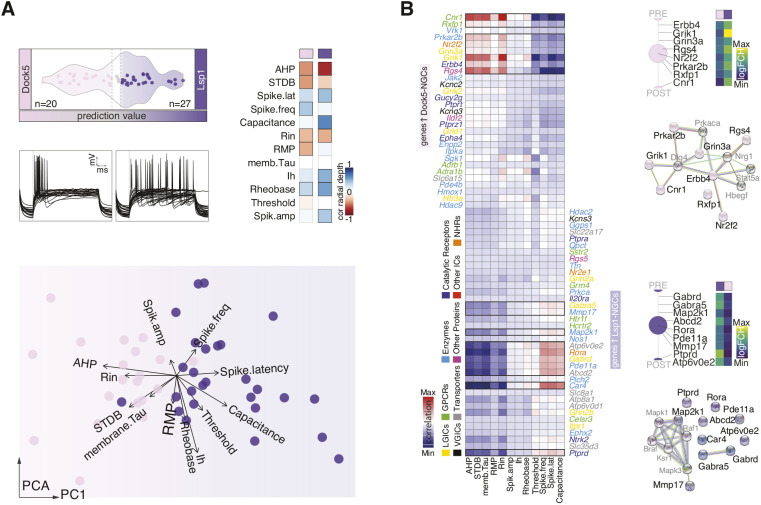
**Functional correlates of NGC subtypes.** (A) Violin plot representing SVM decision values for each NGC collected using patch-seq (*Hmx3*;tdTOM^+^/*Htr3a*-GFP^+^). Color-code indicates SVM-based NGC subtype assignment: Dock5^+^NGC, pink (*n*=20); Lsp1^+^NGC, purple (*n*=27); non-assigned, gray (*n*=3) (Table S9). First-spike electrophysiological traces for each cell are represented and grouped by NGC subtype. Heatmap showing correlation of electrophysiological features respect to radial position in cortex (red, low; white, mid; blue, high) (Table S10). PCA plot calculated on measured electrophysiological features for each single cell and color-coded by NGC subtype) (Table S10). (B) Heatmap representing correlation scores between electrophysiological features and gene expression patterns (SVM genes also contained in the functional IUPHAR database, see Materials and Methods) on patch-sequenced single cells (color gradient: blue, strong anticorrelation; white, no association; blue, strong correlation) (Table S10). Gene names depicted in *y*-axis-left correspond to Dock5^+^NGC-enriched genes and those in *y*-axis-right to Lsp1^+^NGC-enriched genes (SVM model weights, Table S6). Gene name color-coding represents gene categories as annotated in IUPHAR database. Highlighted rows on heatmap signal strongest correlations for NGC subtypes. Subcellular localization (STRINGdb), expression fold change (Viridis color gradient) and predicted protein interaction networks (STRINGdb, including inferred protein partners in gray) are illustrated for strongly correlated genes per NGC subtype (Table S10). AHP, after hyperpolarization potential; amp, amplitude; freq, frequency; Ih, hyperpolarization-activated cation current; lat, latency; Rin, input resistance; RMP, resting membrane potential; STDB, subthreshold depolarizing bump; tau, membrane time constant.

To investigate whether Dock5^+^NGC and Lsp1^+^NGC subtypes have distinct electrophysiological properties, we performed whole-cell electrophysiological recordings followed by scRNA-seq (patch-seq) on fate-mapped NGCs (Fig. S6B, Table S9). Upon applying the previously trained NGC-subtype model (Fig. 2B, Table S6) for predicting their cell type identity (Fig. 3A, Fig. S6A, Table S9), we found that, whereas Lsp1^+^NGCs displayed late-spiking behavior typically associated with NGCs (Schuman et al., 2019), Dock5^+^NGCs were characterized by a fast-spiking profile (Fig. 3A, Table S10). Unsupervised analysis on the array of electrophysiological parameters collected (Fig. S6B, Table S10) revealed that the intrinsic properties of NGC subtypes are differential enough to segregate them linearly (Fig. 3A). This finding suggests that NGC subtypes play distinct roles within cortical circuits as they might respond differently to input stimuli. Specifically, Lsp1^+^NGCs showed a significantly wider after-hyperpolarization potential (AHP) (*P*<0.0001, −14.6±1 for Dock5^+^NGCs, −23.4±0.9 for Lsp1^+^NGCs), together with a longer spike latency (*P*<0.0001, 50.3±5.4 for Dock5^+^NGCs, 205.3.4±25.4 for Lsp1^+^NGCs). Dock5^+^NGCs, instead, displayed a characteristic subthreshold depolarizing bump (STDB) that was absent in most Lsp1^+^NGCs (*P*<0.001, 3.2±0.4 for Dock5^+^NGCs, 0.8±0.4 for Lsp1^+^NGCs; Fig. S6C, Table S10). Moreover, most electrophysiological parameters were correlated with the radial position of the patched neuron, indicating a topographical organization and the existence of physiological gradients within NGCs (Fig. 3A, Fig. S6E, Table S10). For instance, although large STDBs are characteristic of superficially located Dock5^+^NGCs (likely canopy cells; Schuman et al., 2019), DL Lsp1^+^NGCs have wider AHPs and, for both NGC subtypes, spike latency increases with cortical depth (Fig. 3A, Fig. S6E, Table S10). In addition, NGCs populating different cortical layers display distinctive intra-subtype morphologies (reconstructions obtained from Scala et al., 2021) (Fig. S6F). Specifically, irrespective of the NGC subtype, L1 NGCs have horizontally elongated morphologies, whereas non-L1 NGCs display a characteristic oval shape. This is also the case for a third NGC subtype emerging from the *Hmx3* lineage (also *Htr3a*^+^); these cells were very sparse in the neocortex and were here identified using *Nkx2-1* intersectional genetic fate mapping. Found mainly in DLs and likely corresponding to the previously reported *Lamp5*^+^*Lhx6*^+^ subtype (Tasic et al., 2018), examination of the gene expression profiles of these cells revealed communalities with other NGC subtypes, such as the shared expression of *Tox2* (Fig. S6G).

Finally, we examined the extent to which NGC subtype-enriched transcriptional signatures among patched cells were reflective of the observed differential electrophysiological properties. Focusing on genes annotated for their functional relevance (see Materials and Methods), we found that NGC subtype-enriched families of presynaptic ligand-gated ion channels also correlated with cellular intrinsic properties (Fig. 3B, Table S10). Specifically, enrichment of Dock5^+^NGCs in glutamate receptor subunits (i.e. *Grik1*, *Grin3a*) and high expression of GABA receptor subunits (i.e. *Gabrd*, *Gabra5*) in Lsp1^+^NGCs was strongly related to NGC subtype-specific properties, such as AHP, STDB or spike latency.

Together, these findings indicate that NGC subtypes are functionally different, as shown by their electrophysiological properties, and thus might respond differently to input stimuli. Moreover, these distinct intrinsic profiles correlate with NGC subtype expression of enriched synaptic gene families, further suggesting specialized circuit partners and roles in cortical networks.

### NGC embryonic emergence and diversification

**Fig. 4.**
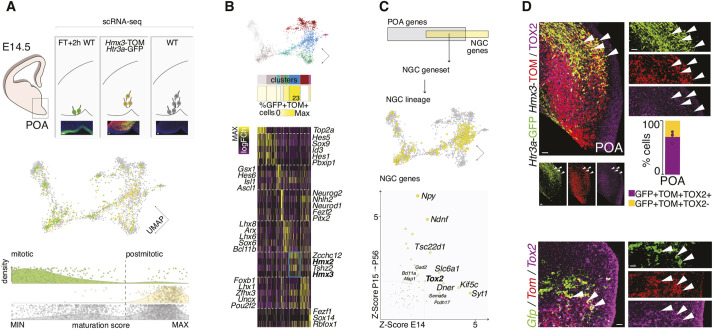
**NGC embryonic emergence and diversification.** (A) Schematic illustrating scRNA-seq populations captured at E14.5 in POA accompanied by microscopy images. Three independent POA datasets were collected: +2 h FlashTag (green, *n*=352 cells), *Hmx3*;tdTOM^+^/*Htr3a*-GFP^+^ (yellow, *n*=418 cells) and WT (gray, *n*=2106 cells) (Table S12). UMAP scatter plot showing result of the POA E14.5 dataset integration (color-coded by capture protocol). Density plots depicting cell distribution along their maturation trajectory (PCA pseudotime, Fig. S8A,E), color-coded and split by dataset. (B) POA-integration UMAP plot color-coded by cluster. Heatmap in shades of yellow shows the percentage of fate-mapped NGCs populating each cluster (Table S13). Heatmap depicting the gene expression log fold change (FC) for transcription factors enriched on each cluster (color gradient: magenta, low; black, mid; yellow, high). (C) Schematic illustrating the feature selection strategy for defining the NGC gene set and lineage (intersection between POA embryonic variable genes, gray; NGC postnatal genes identified postnatally, yellow). UMAP plot highlighting in yellow embryonic POA cells confidently assigned to as NGCs (Table S12). Scatter plot illustrating *z*-scores for the genes belonging to the NGC gene set (gray-yellow scale and size indicate *z*-score value). (D) Top: Representative *Htr3a*-GFP; *Hmx3*-tdTOM POA E14.5 coronal section image, immunostained against TOX2 (magenta). Arrowheads indicate GFP^+^TOM^+^TOX2^+^ cells. Barplot showing TOX2 protein quantification in embryonic NGCs (71.2±4.7% TOX2^+^; TOX2-NGC fraction shown in yellow; *n*=4 brains, 3024 *Hmx3*;tdTOM^+^/*Htr3a*-GFP^+^ cells) (Table S14). Bottom: Representative *Htr3a*-GFP; *Hmx3*-tdTOM E14.5 coronal section image at the level of the POA stained by smFISH against *Tox2* (arrowheads indicate *Gfp*^+^*Tom*^+^*Tox2*^+^ cells). Scale bars: 25 µm (A); 25 µm (D, IHC); 15 µm (D, FISH).

NGCs are diverse both in molecular identity, laminar distribution and functional patterns. We therefore next aimed at examining how NGC molecular heterogeneity emerges during embryonic development. To cover the different early maturation stages of NGC embryonic maturation, we microdissected the embryonic POA at embryonic day (E) 14.5 and collected non-fluorescent POA cells [wild-type (WT) embryos], 2-h-old FlashTag^+^ (FT) POA progenitors (WT embryos) (Telley et al., 2016) and *Hmx3*;tdTOM^+^/*Htr3a*-GFP^+^ post-mitotic NGCs using FACS and performed scRNA-seq (Fig. 4A, Fig. S7, Tables S11, S12).

To understand POA IN embryonic development, we first reconstructed single-cell maturation trajectories by ordering them according to their positions along a principal components analysis (PCA)-fitted principal curve (see Materials and Methods, Fig. S8A, Table S12). This allowed us to identify sequential waves of gene expression across maturation that, together, reflect the early molecular development of INs, including NGCs (Fig. S8D). We found that newborn IN maturation programs are shared between IN cell types; namely, they largely overlap with that of CGE-derived INs. This result aligns with previous reports supporting the existence of a pan-IN early maturation schema (Mayer et al., 2018) (Fig. S8A-D′).

Next, we integrated the three E14.5 POA datasets to generate a uniform manifold approximation and projection (UMAP) on which we performed cluster analysis (see Materials and Methods; Fig. 4A, Table S12). We found eight transcriptional clusters exhibiting TF-specific codes (Fig. 4B, Fig. S8E), one of them corresponding to putative post-mitotic NGCs given its expression of *Hmx2* and *Hmx3* as well as its enrichment in *Hmx3*;tdTOM^+^/*Htr3a*-GFP^+^ cells (23%) (Fig. 4B, Tables S12, S13). To narrow down NGC identification among E14.5 POA single cells, we found 88 NGC time conserved genes by cross-comparing embryonic (E14) and postnatal (P15, P30 and P56) datasets (Fig. 4C, Table S13). This approach highlighted *Tox2* as a TF constitutively and stably expressed in NGCs through development. Supporting this, *Tox2* mRNA and protein expression was detected in POA-located NGCs and was found to be enriched in cells sitting along the POA ventricular wall (dividing progenitors) (Fig. 4D, Table S14). Importantly, *Tox2* transcripts were undetectable in the CGE (Fig. S8H).

Aiming at distinguishing NGC subtypes among post-mitotic NGCs and understanding whether they differ on birthdate and molecular signatures, we used E10- to E14-born NGCs from a recent lineage-tracing dataset (Bandler et al., 2022). To this end, we first predicted NGC subtype identity using previously identified molecular signatures (Fig. 2B, Table S13). This revealed that Lsp1^+^NGCs neurogenesis starts earlier (E10) than for Dock5^+^NGCs (E13), as NGCs born before E13 were never predicted as Dock5^+^NGCs (Fig. S8F). Then, we found differentially expressed genes among NGC post-mitotic subtypes, indicative of embryonic cell type diversification at E14.5, which might instruct these cells to respond differently to microenvironmental signals. An example was the complementary expression of Eph receptors. *Epha4* was enriched in predicted post-mitotic Dock5^+^NGCs, whereas *Epha3* was expressed in Lsp1^+^NGCs.

Together, these findings indicate that, although NGCs overarching early maturation programs are shared with other IN types, NGC-specific programs can be detected at the cycling progenitor stage, as shown by their characteristic expression of *Tox2*. NGC subtype molecular divergence emerges early post-mitotically, as evidenced by the presence of distinct populations of Dock5^+^NGCs and Lsp1^+^NGCs predicted precursors.

### *Tox2* is required for NGC development

**Fig. 5.**
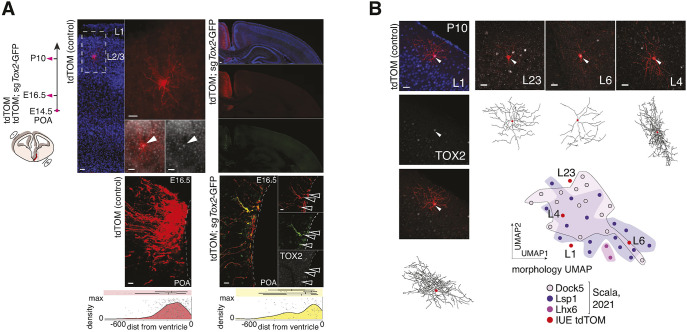
***Tox2* plays a crucial role in NGC development.** (A) Schema illustrating *in utero* electroporation experimental strategy and analysis pipeline for downregulating *Tox2* in POA cells at E14.5 (phenotype analyzed at E16.5 and P10) [plasmids were electroporated at E14.5 (pink circle) in the POA and samples analyzed either at E16.5 or at P10 (pink arrows)]. Low- and high-magnification illustrations of electroporated brains with control (tdTOM, left) or tdTOM;sg*Tox2*GFP (right) at P10 (neocortex; filled arrowheads indicate a Tom^+^ cell) and E16.5 (POA; unfilled arrowheads indicate Tom^+^ Gfp^+^ Tox2^−^ cells) illustrating the decreased number of electroporated cells found in POA at E16.5 upon tdTOM or sg*Tox2* electroporation (*n*=2 control brains, 351 cells, 175±71.5; *n*=4 sg*Tox2* brains, 110 cells, 27.5±4.5). Density/scatter plots illustrating the position of electroporated cells quantified in POA at E16.5 (Table S15) (*x*-axis: cell distance in pixels from the POA ventricular surface; *y*-axis: density kernel with identical limits both for tdTOM and sg*Tox2* experiments). Cell distribution was significantly different depending on experimental condition (D=0.21, *P*-value=0.0007; Kolmogorov–Smirnov two-sided test). Biological replicates split in inset boxplots (red: tdTOM control cells; yellow: tdTOM;sg*Tox2*GFP CRISPR-targeted cells). (B) Morphology reconstructions of control-electroporated (tdTOM) NGCs at P10 located in different cortical layers (L1, L23, L4, L6) together with their TOX2 protein expression (gray) (white arrowheads indicating TOX2 expression in NGC nuclei). UMAP built on morphology feature extraction (Table S15) shows alignment between NGCs reconstructed from tdTOM control electroporations and from previously published data (Scala et al., 2021) (*in utero* electroporation: *n*=2 brains, 4 cells) (Table S15). Scale bars: 50 µm (A, P10 low magnification); 20 µm (A, P10 high magnification); 50 µm (A, P10 hemisphere); 10 µm (A, E16.5 high and low magnification); 25 µm (B).

Having identified *Tox2* as an NGC constitutive transcription factor, we aimed to investigate its role in NGC development. To do so, we downregulated the expression of this TF in POA-born cells at E14.5, the location and time-window in which both NGC subtypes are being generated (Fig. 5A, Fig. S8F). To this end, we used *in utero* electroporation to deliver a CRISPR-Cas9 plasmid (sg*Tox2*GFP) containing three single-guide RNAs against *Tox2* (Fig. S9A), together with a tdTOM-encoding plasmid (used as a control when delivered alone). We validated the sg*Tox2*GFP construct by electroporating a subpopulation of *Tox2*-expressing cortical neurons in deep cortical layers (E12.5) and showed that our plasmid effectively reduced the production of the TOX2 protein (Fig. S9A).

*In utero* electroporation of tdTOM plasmid alone in E14.5 POA progenitors labeled cortical NGCs expressing TOX2 at P10 (Fig. 5B). Morphology reconstructions and their integration into a reference NGC morphology atlas (Scala et al., 2021) indicated that P10 tdTOM-electroporated NGCs were morphologically diverse, putatively belonging to distinct NGC subtypes and positioned in different cortical layers (Fig. 5B, Table S15). Conversely, when the sg*Tox2*GFP plasmid was co-electroporated, we observed a complete depletion of NGCs in the cortex (Fig. 5A). Concomitantly, we observed cell distribution alterations in the hypothalamus at P10, indicating that *Tox2* is instrumental for the proper maturation of other POA-born cell populations (Fig. S9B, Table S15).

To improve our understanding of the developmental processes leading to the observed phenotype, we analyzed *Tox2* downregulation effects at E16.5. The number of electroporated cells populating the embryonic POA 2 days after electroporation was decreased following *Tox2* downregulation (Fig. 5A, Table S15; *n*=2 control brains, 351 cells, 175±71.5; *n*=4 sg*Tox2* brains, 110 cells, 27.5±4.5). Consistently, we observed sg*Tox2*GFP^+^CASP3^+^ reactive cells at E16.5 in the POA that were abnormally located proximal to the ventricular wall, suggesting a defect in migration followed by cell apoptosis (Fig. S9C).

Together, these results indicate that *Tox2* is essential for NGC development and fate acquisition, as POA-born cells lacking *Tox2* fail to differentiate into NGCs.

## DISCUSSION

Here, we examined the emergence of subtype diversity within neocortical NGCs, constituting a genetically traceable and functionally distinctive IN type with an unusual embryonic origin, the POA (Niquille et al., 2018). By mapping the transcriptomic identity of *Hmx3*;tdTOM^+^/*Htr3a*-GFP^+^ UL NGCs (35% of INs in L1; Niquille et al., 2018; Tremblay et al., 2016), we first revealed their subtype heterogeneity. Two main NGC subtypes mainly populated cortical ULs. With segregated laminar distributions, Dock5^+^NGCs were preferentially found in L1, whereas Lsp1^+^NGCs were enriched in L2/3 and could be occasionally found across all cortical layers. A third group, very rare, was found in cortical DLs. These scarce-in-cortex NGCs also express the TF *Tox2* and were found in the embryonic POA but, unlike other NGCs, derive from a mixed *Nkx2-1*/*Hmx3* lineage. They likely correspond to the recently described *Id2*/*Nkx2­-1* cells (Valero et al., 2021), previously named as Lamp5 Lhx6 cells by Tasic et al. (2018).

Next, we identified NGC postnatal time-conserved molecular signatures. The two main NGC subtypes (Dock5^+^NGCs and Lsp1^+^NGCs) expressed complementary gene sets, the functional relevance of which strongly correlated with distinctive electrophysiological profiles. For instance, Lsp1^+^NGCs had an enriched expression of *Npy*, long considered as an NGC maker (Gelman et al., 2009; Ibanez-Sandoval et al., 2011), but lowly expressed in superficial Dock5^+^NGCs. The latter showed enriched expression of *Ndnf* and had molecular and electrophysiological profiles consistent with the recently described ‘canopy’ cells (Schuman et al., 2019; Webster et al., 2021). Furthermore, we identified the relaxin receptor 1 (*Rxfp1*) as specific marker for Dock5^+^NGCs. Although the role of *Rxfp1* in INs remains unknown, a large spectrum of modulatory actions have been associated with the receptors of this family (Gundlach et al., 2009). In addition, UL NGC subtypes display complementary expression of well-described ion channel subunits, suggesting their distinct involvement in cortical regulation and specialized synaptic input partners. Molecularly distinct NGC subtypes were detectable as early as in their embryonic region of origin. Post-mitotically, NGC subtypes displayed marked molecular differences, potentially suggesting that they are tuned for divergent interactions with the surrounding environment (i.e. differential expression of Eph receptor transcripts).

Differences found at the molecular level also reflected discrete electrophysiological patterns. Dock5^+^NGCs displayed a marked STDB, and Lsp1^+^NGCs spiked with a much larger latency and presented a substantially larger AHP, consistent with their higher capacitance and spike frequency. Although we did not address questions regarding their connectivity with other neurons in the neocortex, the differences we found are consistent with recent studies on NGCs populating L1. Indeed, each of these NGC subtypes are able to modulate pyramidal cell distal dendrites and/or selective IN subtypes, conferring them a specific output and thus modulatory properties in the circuit (Genescu and Garel, 2021; Hartung and Letzkus, 2021). Furthermore, at least in L1, a plethora of long-range axons are present (e.g. serotonergic, cholinergic, thalamocortical) and NGCs subtypes could be specialized in gathering and propagating information from specific modulatory inputs (Cocas et al., 2016; Gesuita and Karayannis, 2021; Schuman et al., 2021).

By labeling NGCs in the POA at birth by *in utero* electroporation and crossing subtype predictions with lineage-tracing evidence (Bandler et al., 2022), we showed that these cells are produced between E10 and E15, as suggested by previous pioneer studies (Gelman et al., 2009). In addition, we saw that Lsp1^+^NGCs appear to be generated before Dock5^+^NGCs. However, here we did not address the question of whether the generation of NGC subtypes is consequent to microspatial factors or cell cycle dynamics, as is the case for MGE-born IN subtypes (Lim et al., 2018; Petros et al., 2015), or, alternatively, is determined in a post-mitotic state (Mayer et al., 2018).

In a quest for genes that could characterize NGCs from birth to cortical integration, we discovered that, independently of the subtypes we have identified, all NGCs shared a protracted and specific expression of *Tox2*. This TF is likely a hallmark for NGC identity emergence and/or maintenance compared with other *Htr3a*^+^ INs. Among all IN subtypes, NGCs express the highest levels of *Tox2*, whereas some *Pvalb*^+^, *Sst*^+^ and L5 excitatory neurons express it at lower levels. Downregulation of *Tox2* in the POA using *in utero* electroporation at E14.5 suggested that it plays a crucial role in NGC development, because POA-born cells lacking *Tox2* either die or fail to differentiate into NGCs. Roles for *Tox2* have been previously reported in immunology, where it is described as a regulator of T-cell proliferation, specification and maturation (Aliahmad et al., 2011; Seehus et al., 2015; Veldman et al., 2022; Xu et al., 2019). Moreover, in genome-wide association studies, *Tox2* has been identified as deregulated in psychiatric conditions (Dick et al., 2011; Edwards et al., 2012), but investigations into its role in interneurons have never been addressed. The TOX transcription factor family is composed of four members, *Tox*, *Tox2*, *Tox3* and *Tox4*, which are expressed combinatorially in different IN subsets (celltypes.brain-map.org/rnaseq). There is thus potential for coordinated action and/or cross-regulation mechanisms between TOX members for IN cell-type specification. Supporting this possibility, evidence from immunology research suggests that coordinated action between Tox and Tox2 is necessary to upregulate PD-1 (PDCD1) expression (Seo et al., 2019).

Overall, we shed light on the subtype diversity within cortical *Hmx3*;tdTOM^+^/*Htr3a*-GFP^+^ NGCs, which exhibit distinct molecular, morphological and electrophysiological features. Although NGC subtype molecular differences appear to emerge gradually through development, all the NGCs studied here are rooted in the expression of a shared TF, *Tox2*. This work provides a basis for further and thorough interrogations of the functional relevance of the distinct NGC subtypes as part of a complex cortical circuit and as contributors and modulators of behavioral states.

## MATERIALS AND METHODS

### Mouse strains

Animal experiments were performed according to international and Swiss guidelines and approved by the Geneva local animal care committee. Timed-pregnant transgenic females were obtained by overnight mating. Mice were maintained on a C57BL/6 background and both female and male embryos and mice were analyzed in this study. CD1 mice used for experiments involving embryonic Flash-Tag injections, *in utero* electroporations or for droplet-based scRNA-seq were purchased from Charles River Laboratories and E0.5 (overnight-mated females) was established as the time of detection of the vaginal plug. Mice were housed in the conventional area of the animal facility of the University Medical Center, under controlled temperature (22±2°C) and dark/light cycles (12 h each). Food and water were provided *ad libitum*. We crossed B6.Cg-*Gt(ROSA)26Sor^tm14(CAG-tdTomato)Hze^*/J (The Jackson Laboratory, stock #007914) with Tg(*Htr3a*-EGFP)DH30Gsat/Mmnc (GENSAT Consortium) bacterial artificial chromosome transgenic (BAC) mice to obtain *Htr3a*-GFP; Ai14 (*Rosa26*-tdTOM^fl/fl^) mice. *Gfp* expression levels in the *Htr3a*-GFP BAC transgenic line are highly correlated with the expression levels of the *Htr3a* gene (see Data availability section). To label NGCs, these mice were then crossed with Tg(*Hmx3*-icre)1Kess BAC transgenic line (also known as *Nkx5.1*-Cre) animals to obtain the *Hmx3*- Cre::*Htr3a*-GFP;Ai14 mouse model reported by Niquille et al. (2018). *Tom* expression levels in the *Hmx3*-Cre;Ai14 line recapitulate Cre expression levels (*Hmx3* Cre-mediated recombination happens embryonically, *Hmx3* is not expressed postnatally). Both *Htr3a*-Gfp and the *Hmx3*-Cre are BAC transgenic lines, which, although highly stable and with a low probability of chimerism, could affect endogenous gene expression (Gong et al., 2007). To elucidate whether NGC progenitors belong to an *Nkx2-1*^+^ lineage, *Hmx3*-cre animals were first bred to *Nkx2-1^tm2.1(flpo)Zjh^*/J (The Jackson Laboratory, stock #028577) and finally crossed with B6;129S4- Gt(ROSA)26Sor^tm3(CAG-tdTomato,-EGFP*)Zjh^/J (IS reporter; The Jackson Laboratory, stock #028582) (He et al., 2016).

### Surgical procedures

Overnight-mated CD1 pregnant dams (age of embryos: E14.5) were used for *in utero* experiments: Flash Tag (FT) injections and *in utero* electroporations. One hour before surgery, pregnant CD1 dams were provided with analgesia (subcutaneous Temgesic 0.1 mg/kg, Schering-Plough) and anesthetized by inhalation of isoflurane (2.5%, Baxter). Mice were placed on a sterilized surgery table (temperature controlled at 37°C), their eyes protected with gel drops (Viscotears), and their abdomen shaved, sterilized with Betadine (MundiPharma) and covered with a sterile pad. During the surgical procedure (*in utero* FT or electroporation), uterine horns were exposed by cesarean cut along the linea alba and embryos were kept moisturized by continuous application of warm 0.9% NaCl. Once the surgical procedure completed, a second identical dose of Temgesic was administered prior to the abdominal wall being closed. Mice were monitored while recovering on a warm pad for 2 h post-surgery before being placed in the animal house.

For experiments involving FT injections, embryos were allowed to develop for 2 h prior to tissue collection in order for progenitors located in the embryonic brain's third ventricle wall to be stained.

#### Flash tag *in utero* injection

Half a microliter of 10 mM of a carboxyfluorescein succinimidyl ester (Flash Tag, CellTraceTM CFSE, Life Technologies, C34554) was injected into the lateral ventricle of the embryo's brain through a beveled glass pipette (Drummond Scientific) coupled to a Picospritzer (Parker).

#### *In utero* electroporation

Ten microliters of 2 μg/ μl (with 1% Fast Green, Sigma-Aldrich) plasmids pCAG-IRES-tdTOM (Addgene #83029) and Ef1a-sg*Tox2*Cas9-2A-GFP (purchased from abm, 473231140591; subcloned, transformed and amplified following manufacturer's protocol), in equal proportions or the first alone, were injected as for FT. Tweezer-type electrodes (CUY611P3-1, NepaGene) were placed on the embryo's brain at an appropriate angle to target electrically the POA. Five square pulses of 45 V (50 ms on/950 ms off) for E14.5 and five square pulses of 35 V (50 ms on/950 ms off) for E12.5 electroporations were applied with a square wave electroporator (ECM830, Harvard Apparatus). Embryos were let to develop until the age of interest (E16.5 or P10).

### Histology and analysis

#### Tissue preparation

For postnatal ages, mice were euthanized with lethal intraperitoneal injection of pentobarbital (50 mg/kg) and transcardially perfused with 0.9% saline solution with Liquemine (2 ml/l) followed by ice-cold 4% paraformaldehyde (PFA) dissolved in PBS. Brains were dissected and post-fixed overnight at 4°C in 4% PFA under agitation. For embryonic ages, pregnant females were euthanized with lethal intraperitoneal injection of pentobarbital and embryos exposed by cesarian cut. Brains were dissected in ice-cold PBS and post-fixed overnight at 4°C in 4% PFA under agitation. For smFISH, after overnight fixation both postnatal and embryonic brains were soaked in increasing percentages of sucrose solution (15% and 30%; diluted in PBS; Sigma-Aldrich). When sunk, brains were then placed in O.C.T Compound (Tissue-Tek, 4583) then in isopentan (2-metylbutane, ReagentPlus, ≥99%; Sigma) and placed on dry ice for quick freezing. Brain cubes were stored at −20°C until processed.

#### smFISH

Coronal sections (12 µm thick) from embryonic samples were prepared using a cryostat (Leica CM3050) and mounted on microscopy slides (Superfrost Plus; Thermo Fisher Scientific). Slides were stored at −80°C after drying if not immediately processed. Prior to hybridization, sections were fixed with 4% PFA for 15 min and processed for staining according to the manufacturer's instructions, using the RNA-scope Multiplex Fluorescent Reagent Kit v2 Assay (Advanced Cell Diagnostics) for fixed frozen tissue. Briefly, sections were dehydrated using 50%, 70% and 100% successive baths. A 10 min treatment in SDS (4% in 200 mM sodium borate) was added to the protocol after the Protease IV incubation as proposed by Zeisel et al. (2015). RNA-Scope Probes (Advanced Cell Diagnostics, Table S16) were then incubated on sections for 2 h at 40°C and processed for amplification steps. Finally, sections were counterstained with Hoechst 33258 (Sigma-Aldrich) for 10 min, mounted with Mowiol medium (Merck, 9002-89-5) and left to dry overnight before imaging.

#### Immunohistochemistry

Brains were sliced coronally at 70 µm using a vibratome (VT100S, Leica) and stored at −20°C in an ethylene-glycol-based cryoprotective solution if not immediately processed. Brain slices were permeabilized in 0.3% Triton X-100 and 0.02% sodium azide in PBS and blocked in 2% normal horse serum for 2 h and incubated with primary antibodies overnight (Table S16). Slices were finally incubated with appropriate secondary antibodies, counterstained with Hoechst 33258 (Sigma-Aldrich) and mounted using Mowiol medium.

#### Imaging

Fluorescent images were acquired using either confocal (channel colocalization and 3D stacks) or widefield (single channel) microscopes. Confocal systems used were Nikon A1r (20×0.45 CFI ELWD Plan Fluor or 40×0.6 CFI ELWD Plan Fluor objectives) and LSM800 Airyscan (40×1.4 Oil DIC Plan-APO objective); widefield imaging was performed using the Widefield scanner Zeiss Axioscan Z1 (20×0.8 Plan Apochromat objective).

#### Image preprocessing, cell quantification, morphological reconstruction and analysis

For smFISH experiments aiming at obtaining single-molecule quantitative resolution (Fig. 2A,B), custom MATLAB scripts (Geneva University Bioimaging Core Facility) were used for signal preprocessing, detection and quantification. For histological preparations aiming at single-cell quantitative resolution (Figs 1B, 4D, 5, Figs S6, S8, S9B,C), images were preprocessed and single cells counted using Fiji software, whereas analysis (frequencies, positioning) was performed using custom R scripts. Differences in cell distributions were assessed using two-sided Kolmogorov–Smirnov tests (R stats) (Figs 1B, 5B, Fig. S9B) on reference-corrected cell positions (pia surface for cortex, third ventricle for POA and hypothalamus). Morphological reconstructions of NGCs imaged as high-resolution 3D confocal stacks were semi-automated using neuTube 1.0z (Feng et al., 2015), exported in SWC format and visualized using nat (R) (Jefferis and Manton, 2014). Feature extraction on scaled reconstructions was performed using NeuroM (Python) (Palacios et al., 2022).

For addressing population distributions, images were rotated using Fiji for homogeneity across acquisitions: specifically, cortical images were rotated such that the pia surface was the upper-horizontal limit; for POA or hypothalamic slices, the wall of the third ventricle was positioned as the right-vertical limit. Using the Fiji ROI manager plugin, an oval region of interest was drawn on each cell body. Visual inspection was used to determine whether each delimited cell expressed the markers of interest and were binarily encoded by the marker. ROI manager coordinates for each cell and for the pia or ventricular limits as well as single-cell marker binary scoring were analyzed using custom R scripts aiming at calculating population percentages and density estimates on single-cell radial position (normalized to the pia surface or the third ventricle). The coordinate axis not subjected to normalization (*x* in the cortex and *y* in the POA or hypothalamus) was jittered according to a scaled interval within the region limits.

#### IUE morphology UMAP and cell type identity prediction

Feature extraction datasets (NeuroM) from (1) Scala et al. (2021) NGCs and (2) morphologically reconstructed NGCs obtained from control POA E14.5 *in utero* electroporation experiments (pCAG-tdTOM) were integrated in Seurat v3. This morphological integration space was used for predicting subtype identity of IUE-reconstructed cells by label transfer.

### scRNA-seq collection, sequencing, mapping and counting

All single-cell RNA capture, library preparation and sequencing procedures were performed within the Geneva University Genomics Core Facility. Two scRNA-seq methods were used: microfluidic-based scRNA-seq for experiments involving FACS (P15, P30 and E14.5 *Hmx3*-Cre::*Htr3a*-GFP; *R26R*-tdTOM^fl/fl^ ; E14.5 Flash Tag) and droplet-based for experiments on WT cells (E14.5).

#### Microfluidic based scRNA-seq

For postnatal tissue dissociation, P15 and P30 *Hmx3*-Cre::*Htr3a*-GFP; *R26R*-tdTOM^fl/fl^ brains were extracted in ice cold Hanks' balanced salt solution (HBSS; Sigma-Aldrich) and coronal slices (600 µm) were cut using a Mcllwain tissue chopper. Upper layers of somatosensory cortex were microdissected. Each time point consisted of pooled brains (*n*=5 at P15 and *n*=6 at P30). For tissue digestion, a modified protocol for the Worthington Papain Dissociation kit (Worthington Biochemical Corporation, LK003150) was used. Tissue was placed in EBSS#1 solution complemented with AP5 (0.05 mM, Tocris, 0106), kynurenic acid (0.8 mM, Sigma-Aldrich, K3375) and trehalose (0.135 M, Sigma-Aldrich, T9531) and then transferred to a papain bath for 15 or 30 min at 37°C under gentle agitation for P15 and P30, respectively. Trituration with a 1 ml pipette was performed and the obtained cloudy cell suspension was centrifuged at 300 ***g*** for 5 min. The pellet was resuspended with 3 ml of EBSS#2 solution complemented with AP5 (0.05 mM), kynurenic acid (0.8 mM), 350 µl of ovomucoid and 250 µl of DNase and trehalose (0.135 M). After adding the suspension to 5 ml of ovomucoid solution, the mixture was centrifuged at 70 ***g*** for 6 min. The final pellet was resuspended in 1 ml of DMEM/F12 complemented with 10% fetal bovine serum, 10% horse serum, AP5 (0.025 mM), kynurenic acid (0.4 mM) and trehalose (0.135 M). Finally, cells were incubated with Hoechst 33342 (1 µg/ml, Sigma-Aldrich, H1399) for 15 min at 37°C and FACS-sorted using a Beckman Coulter MoFlo Astrios set for selecting GFP^+^ cells on one side and GFP^+^/Tomato^+^ cells on the other side. A mix of 1 µl of Cell Suspension Reagent (Fluidigm/Standard BioTools) and 9 µl of each of the two FACS-sorted cell suspensions (500 cells/µl) was loaded on a C1 Single-Cell AutoPrep integrated fluidic circuit (IFC) designed for 10-17 µm cells (HT-800, Fluidigm/Standard BioTools, 100-57-80). Immediately after the single-cell capture, the IFC plate was imaged under two different filters (GFP 3035B and Cy3 4040B) in addition to the brightfield using the ImageXpress Micro Widefield High Content Screening System (Molecular Devices). For embryonic tissue dissociation, either WT E14.5, E14.5+2 h FT-injected brains or E14.5 brains from *Hmx3*-Cre::*Htr3a*-GFP; *R26R*-tdTOM^fl/fl^ mice were used. Embryonic brains were extracted in ice-cold HBSS, and the POA or CGE was microdissected and incubated in 0.05% trypsin at 37°C for 5 min. After mechanical dissociation, cells were centrifuged for 5 min at 300 ***g*** and the pellet was suspended in 1 ml of HBSS then through a 70 µm cell strainer. FT^+^ cells were FACS-sorted on a MoFlo Astrios device (Beckman Coulter) gated to include only the top 5% brightest cells. *Hmx3*-Cre; tdTOM^+^; *Htr3a*-GFP^+^ POA cells were FACS-sorted on a MoFlo Astrios device for selecting GFP^+^/Tomato^+^ cells.

Lysis, cDNA synthesis and pre-amplification steps were performed on the C1 instrument according to the manufacturer's protocol using the SMARTer Ultra Low RNA kit (Takara Bio, 635026). For each IFC, 20 libraries were prepared using a Nextera XT DNA Library Preparation Kit (Illumina, FC-131-1096), multiplexed and sequenced in paired-end mode consisting of a 5 bp unique molecular identifier (UMI) on read 1 and 90 bp on read 2 using a HiSeq2500 instrument (Illumina) to an expected depth of 1 M reads per cell. Sequenced reads were aligned to the mouse genome (GRCm38) using the read-mapping algorithm STAR (Dobin et al., 2013). UMIs were used to correct for cDNA PCR amplification biases. Duplicated reads were identified and corrected using the deduplication step from the UMI-tools software (Smith et al., 2017). Non-ambiguously mapped exonic reads (STAR mapping quality ≥255) were quantified using the ‘summarizeOverlaps’ function from the GenomicAlignments R-Package (mode IntersectionStrict) considering their mapping strand. Unmapped reads were further aligned onto eGFP and Wpre-TdTomato sequences to identify *Htr3a*-GFP and *Hmx3*-Cre;tdTOM positive cells, respectively. This transcriptomic information was cross-compared with fluorescence levels observed after IFC plate picture annotation.

#### Droplet-based scRNA-seq

E14.5 C57BL/6 WT embryos were used for droplet-based RNA-seq. Tissue dissociation was performed as previously described for microfluidic embryonic preparation. Cell suspension was loaded into a 10x Chromium Controller (10x Genomics) and processed with the Single Cell 3′ v2 reagent kit (10x Genomics) according to the manufacturer's protocol. Briefly, single cells were partitioned into gel beads in emulsion (GEMs) in a 10x Genomics GemCode instrument followed by cell lysis and barcoded reverse transcription of RNA, amplification, shearing and 5′ adaptor and sample index attachment. For the POA and CGE E14.5 libraries, 4978 and 2736 cells were recovered, respectively, after being sequenced on a HiSeq 4000 instrument (Illumina) at an expected depth of 70,000 reads per single cell. ‘Cell Ranger’ software (10x Genomics, version 3.0.2) was used for mapping reads to the mouse genome provided by the instrument manufacturer (10x Genomics, mm10 refdata v3.0.0) and for generating feature-barcode matrices.

### Single-cell patch-seq

Dissected *Hmx3*-Cre::*Htr3a*-GFP; *R26R*-tdTOM^fl/fl^ brains (from P14 to P25) were immediately transferred into ice-cold sucrose cutting solution equilibrated with 95% O_2_ and 5% CO_2_ containing (in mM) sucrose (75), NaCl (85), CaCl_2_ (0.5), MgCl_2_ (4), NaHCO_3_ (24), KCl (2.5), NaH_2_PO_4_ (1.25) and glucose (25). A Leica VT 1200S vibratome was used to obtain 300-μm-thick coronal slices, which were then transferred and incubated at 35°C for 20 min in a slice recovery chamber filled with artificial cerebrospinal fluid (ACSF) containing (in mM) NaCl (125), CaCl_2_ (2.5), MgCl_2_ (1), NaHCO_3_ (26), KCl (2.5), NaH_2_PO_4_ (1.25) and glucose (25). For recording, slices were continuously superfused with oxygenated ACSF maintained at 30±0.3°C using an in-line heating system (TC-01, Multi Channel Systems). *Hmx3*; tdTOM^+^ / *Htr3a-*GFP^+^ neurons in cortical layers 1-6 were visualized using an upright microscope (BX51WIF, Olympus), equipped with a 40× water-immersion objective, infrared/differential interference contrast (DIC) optics and epifluorescence (GFP and mCherry filter set and two single fixed wavelengths; LED sources: 470 nm and 565 nm, COO-LED2LLG-470-565, CoolLED). Neurons were digitally visualized using a CCD camera system attached to BX51WIF (SciCam Pro CCD camera, Scientifica). Autoclaved borosilicate glass capillaries (1.5 mm OD, GC150TF-7.5, Harvard Instruments) were used to pull recording pipettes with resistance between 2-4 MΩ using Zeitz DMZ puller (Zeitz-Instruments). Pipettes were filled (up to 1 µl) with RNase-free internal solution containing (in mM): potassium gluconate (123), KCl (12), HEPES (10), EGTA (0.2), MgATP (4), NaGTP (0.3), sodium phosphocreatine (10), 20 μg/ml glycogen and 0.4 U/μl recombinant RNase inhibitor (Takara Bio, 2313A), pH ∼7.3.

Once a GΩ seal was established, neuronal membrane was ruptured with mild negative pressure to enter in the whole-cell configuration. Whole-cell recordings were acquired using a Multiclamp 700B amplifier (Molecular Devices) and digitized at 10 kHz (National Instruments) using a custom-written script in Igor Pro (WaveMetrics). After break-in, the capacitive transients were compensated and capacitance values were recorded from the Multiclamp 700B commander. Cells were held at −70 mV in voltage-clamp mode and a repetitive pulse of −4 mV was given 0.1 Hz to monitor series resistance (R_s_). Neurons with a stable R_s_ and a stable resting membrane potential below −60 mV were subjected to a battery of current injection protocols to study electrophysiological properties, namely input resistance (R_input_), action potential (AP) properties, and sag ratio. For computing R_input_, −40 pA pulse for 120 ms was given and R_input_ values were calculated using Ohm's law. AP properties were studied by delivering consecutive current pulses, 500 ms duration each, from +5 to +300 pA with a 5 pA increment. For sag calculation, a hyperpolarizing current injection step of −200 pA for 500 ms was delivered. This current injection protocol (sweep) was repeated up to ten times and averaged traces were used for data analysis.

#### Electrophysiology analysis

Neurons with >25 MΩ initial R_s_ or fluctuation of>20% during recording were excluded from the analysis. Offline analysis of electrophysiological data was carried out using Igor Pro (WaveMetric), several electrophysiological parameters were manually computed. The STDB was measured as a small depolarization exhibited at sub-threshold current injection. The first AP elicited in response to threshold depolarizing current injection was used to calculate the single AP parameters. The AP train elicited in response to the current injection of +300 pA for 1000 ms was used for calculation of spike frequency and other AP train parameters. The membrane time constant (τ) was computed by monoexponential fit to the first 100 ms after current injection of −40 pA. The sag ratio was calculated using the equation (V_min_ – V_end_)/V_min_, where V_min_ is the minimum voltage reached during the hyperpolarizing pulse of −200 pA, and V_end_ is the final voltage reached at end of current injection. For first spike isolation, the Allen SDK Anaconda environment with Python version 3.7 was used for R reticulate calling of the ‘allensdk.ephys.epys_extractor’ function.

### scRNA-seq analysis

#### Quality control for microfluidic-based datasets

Doublet cells or empty wells identified on the Fluidigm C1 plate imaging were excluded for analysis. At P15 and P30 time points, cells expressing <1000 genes or <100.000 UMIs or <50.000 mapped reads or >20% of reads from the mitochondrial genome were also excluded from the analysis (Fig. S1A,B). At E14.5 time point, C1 cells expressing <1000 genes or <100.000 UMIs or <50.000 mapped reads or >15% reads from the mitochondrial genome were excluded from the analysis (Fig. S7) and non-GABAergic populations were filtered by clustering on 2000 most variable genes (9.7% among E14.5 *Hmx3*-dtTOM^+^; *Htr3a*-GFP^+^ cells and 15.7% among FT^+^ cells). A total of 915 *Hmx3*-dtTOM^+^; *Htr3a*-GFP^+^ single cells (E14.5: 418; P15: 196 cells; P30: 301 cells), 474 *Hmx3*-dtTOM^−^; *Htr3a*-GFP^+^ (P15: 194 cells; P30: 280 cells) and 352 E14.5 FT^+^ cells were kept for further analysis (Figs S1, S7D-G).

#### Quality control on droplet-based datasets

We considered filtered cells from the ‘Cell Ranger' output, and additionally discarded cells expressing <1000 genes or >15% reads from the mitochondrial genome. We additionally identified by clustering on 2000 variable genes and filtered cells expressing known markers of vascular or endothelial (0.62%), red-blood (2.96%), Cajal–Retzius (3.40%), immune (0.93%) and glutamatergic (11.10%) cell types in order to remove non-GABAergic cell populations. The remaining 6221 GABAergic cells (POA: 2106 cells; CGE: 4115 cells) were used for further analysis (Fig. S7A-C).

#### Postnatal maturation reconstruction

P15 and P30 core cells as well as an equivalent number of P56 reference cells were used to train a regularized ordinal regression model to order them on a quantitative maturation score. Inspired by the approach used by Telley et al. (2019), a 10-fold cross-validated linear model was trained using a small set of variable genes for each time point. Prediction weights allowed single cells to be ordered as a continuum in accordance with their developmental maturation stage.

#### Cell type assignment on postnatal datasets

In order to assign a cell type identity to P15 and P30 *Hmx3*;tdTOM^+^/*Htr3a*-GFP^+^ and *Hmx3*;tdTOM^−^/*Htr3a*-GFP^+^ cells that passed quality control criteria (Fig. S1A,B), we used as reference *Htr3a*^+^ core single-cell transcriptomes from the Tasic et al. (2018) adult dataset (*n*=4743, classes: *Lamp5*, *Vip*, *Sncg* and *Serpinf1*). A canonical correlation-based integration (Seurat v2 R package) pipeline was used to project cells in a common t-distributed stochastic neighbor embedding (tSNE) space (independent integrations, P15 Tasic et al., 2018; P30 Tasic et al., 2018) (Fig. 1A, Figs S2A, S3A,B). Label-transfer (cell type assignment) was stablished as the consensus decision between k-nearest neighbors (FNN R package) (on integration tSNE coordinates) and SVM (bmrm R package) classifications (Figs S2B, S3A-B, Tables S1, S2). Cells with divergent cell type assignments across classifiers were considered as ‘inconclusive’ and excluded for further analysis (Table S1), as well as those cells with inconsistent cell type and fluorescence profiles (kept cells were labeled as ‘core’; Table S4).

#### Identification of NGC type and subtype molecular architectures

Consensus predicted core P15 and P30 cells (*n*=158 and *n*=207, respectively), together with a subtype-balanced number of adult cells from Tasic et al. (2018) (*n*=186) were used for training two independent SVM classifiers aiming at identifying postnatally conserved genes for NGC type and non-NGCs [Dock5^+^NGCs and Lsp1^+^NGCs (*n*=270; 69 P15, 111 P30, 90 adult) versus other *Htr3a*^+^ subtypes (*n*=281; 89 P15, 96 P30, 96 adult)] and NGC subtypes [Dock5^+^NGCs (*n*=168; 36 P15, 76 P30, 56 adult) versus Lsp1^+^NGCs (*n*=102; 33 P15, 76 P30, 56 adult)] (Table S4). To ensure that classifiers captured age-conserved genes, cell age was regressed out. All data were used for training and testing to obtain overfitted models for feature selection. The genes identified through classification (150 per class, 300 per classifier) were ranked according to model weights and Log1pRPM fold change (FC) was calculated (Table S5) (for display, FCs were rescaled from −0.5 to 0.5 and single cells were ordered following a gene expression maturation score obtained through ordinal regression using bmrm R package) (Fig. 2A,B).

#### Postnatal gene enrichment analysis

The functional relevance of molecular architecture genes obtained through SVM modelling of postnatal data (NGCs, other *Htr3a*^+^INs, Dock5^+^NGCs versus Lsp1^+^NGCs) (Tables S5, S6) was assessed by gene ontology (GO) term and HUGO Gene Nomenclature Committee (HGNC) gene family enrichment analysis (Figs S4, S5). Specifically, the Mouse Genomic Informatics (MGI) database (http://www.informatics.jax.org/downloads/reports/gene_association.mgi.gz) was used for GO term analysis with its corresponding GO (release 2018-12-28; http://purl.obolibrary.org/obo/go/go-basic.obo) to retrieve enriched GO term ancestors. Similarly, HGNC enriched gene families were identified using the MGI mouse homologs for the HGNC database (release 2018, https://ftp.ebi.ac.uk/pub/databases/genenames/hgnc/tsv/hgnc_complete_set.txt). Hypergeometric tests were used to assess for enrichment compared with the universe of expressed genes across all *Htr3a*-expressing interneuron subtypes. Significant HGNC associations were regrouped into overarching functional families for simplicity of representation (Figs S4C, S5C). A detailed view on HGNC family grouping, GO and HGNC enrichments is available in Tables S5 and S6.

#### Embryonic maturation reconstruction

Embryonic maturation trajectories for E14.5 single-cell datasets were calculated following methods previously described by Telley et al. (2016). All E14.5 single-cell datasets were normalized and scaled regressing for number of genes expressed in order to remove sequencing depth biases. Variable genes common to the different datasets (*n*=675) were identified using the ‘FindVariableGenes’ function from the Seurat R package with default parameters. Data dimensionality was reduced using PCA and only the principal components explaining at least 3% of the data variance were kept. A principal curve was fitted on significant principal components and its orientation determined by the expression of *Nes* and *Dcx*. A maturation score value was attributed to each single cell according to their position when projected along the principal curve and scaled between 0 and 1. Mitotic-to-postmitotic transition was determined by fitting a smooth curve (loess, span=0.25, degree=1) along the coordinates of S-G2/M to maturation score and setting a threshold at the point where the curve falls below the S-G2/M half average.

#### Integration and characterization of POA embryonic datasets

POA-derived E14.5 single cells collected using droplet-based 10x technology (*n*=2106) were used as reference for the integration of the two population-restricted datasets using microfluidic-based C1 technology: (1) E14.5 fate-mapped *Hmx3*-tdTOM^+^/*Htr3a*-GFP^+^ cells (*n*=418) and (2) E14.5 FT^+^ POA-derived progenitors (*n*=352). For the simultaneous integration of three datasets (+2 h FlashTag, *Hmx3*;tdTOM^+^/*Htr3a*-GFP^+^ and WT), we used the Seurat v3 R pipeline. Briefly, E14.5 cells that passed quality control criteria (Fig. S7) were used and each dataset was log-normalized and integration anchors were calculated with default parameters using the union of the 2000 most variable genes for each dataset. Datasets were integrated using 20 principal components and 20 neighbors, scaled and a 2D UMAP was used for representation and clustering using a Seurat standard pipeline.

Markers for each cluster were identified using the ‘FindAllMarkers’ Seurat function and most differentially expressed transcription factors per cluster were illustrated.

For calculating cluster enrichment of *Hmx3*-tdTOM^+^/*Htr3a*-GFP^+^ cells accounting for biases on cluster size, we calculated a seeded random sample of 95 cells per cluster (sample size determined according to the smallest cluster) for assessing the percentage of *Hmx3*-tdTOM^+^/*Htr3a*-GFP^+^ integrated cells by POA population.

#### Identification of NGC-conserved markers and embryonic pseudogene scoring and thresholding

For identifying the genes that characterize NGC cells across development (from progenitors to adulthood), we intersected postnatally conserved NGC versus non-NGC SVM-identified genes respect to all genes expressed in the embryonic POA (>0.05 logRPM). NGC pseudogene was calculated by mean gene expression of NGC-conserved markers normalized to the number of genes expressed in each embryonic cell. The threshold for assignment of embryonic POA cells to the NGC lineage was set at the 80th percentile of NGC pseudogenes across POA embryonic cells. For assessing the likelihood of each gene in the NGC pseudogene to be developmentally conserved, we calculated its *z*-score both embryonically and postnatally with respect to non-NGC cells (*Htr3a*-expressing INs postnatally and POA cells with an NGC pseudogene score below the previously described 80th percentile cutoff).

#### Integration of NGC datasets with STICR-lineage data and subtype embryonic prediction

A freely available STICR-lineage dataset (Bandler et al., 2022) annotated as belonging to the NGC lineage was further annotated by subtype identity using data from Tasic et al. (2018) for Dock5/Lsp1/Lhx6 prediction using the Seurat v3 standard pipeline for integration and label-transfer. E14.5, P15, P30 and P56 (from Tasic et al., 2018) NGCs were integrated into the transcriptomic space of Bandler et al. (2022) NGCs (as reference) for obtaining a cross-dataset NGC subtype landscape and predicting E14.5 NGC subtype identity using the Seurat v3 integration pipeline and label-transfer. The percentage of cells from the Bandler et al. (2022) dataset according to their birthdate annotation was calculated per NGC subtype to assess NGC subtype neurogenesis rates.

#### Embryonic NGC subtypes gene enrichment

The Seurat function ‘FindMarkers' was used to calculate differentially expressed genes between Dock5 and Lsp1 E14.5 NGCs. A pseudotime axis was regressed for discovering embryonic NGC markers not dependent on maturation.

### Patch-seq bioinformatic analysis

#### Quality control on patch-seq cells

The procedure applied to patch-seq dataset sequencing was identical to that applied for postnatal datasets. Quality control determined cells as valid if meeting the following criteria: imaging confirming the cell to be *Hmx3*; tdTOM^+^/*Htr3a*-GFP^+^, containing at least 10,000 sequenced reads, out of those 25% exonically mapped and with <15% of microchondrial reads.

#### Cell type assignment

An SVM model trained for Dock5/Lsp1 categorization was used for predicting cell type identity of patch-seq cells. For this purpose, of the out of the 300 genes found to characterize NGC subtypes, only those that were expressed at least 10% patched cells (*n*=237) were kept. Because of dropout events, patched cells that expressed <20% of the 237 selected genes were discarded. Cell type assignment was annotated for cells for which SVM prediction weight was >0.2. (94%, *n*=47).

#### Electrophysiological PCA and biplot

Electrophysiological feature extraction dataframe and manually annotated features were scaled and used as input for PCA using the ‘prcomp’ R function. R function ‘ggbiplot’ from the R package with identical name was used to draw a biplot indicating the PCA eigenvectors associated with electrophysiological features.

#### Gene-electrophysiological correlates

SVM Dock5/Lsp1-NGCs genes contained in the IUPHAR database (Harding et al., 2022) were used for calculating correlations (Pearson, R) between gene expression and electrophysiological measurements for each patch-seq cell. Significantly correlated genes for each NGC subtype were separately inputted to string-db.org multi protein network inference software using whole *Mus musculus* genome as reference for significance assessment. Correlations between the different electrophysiological measurements were also calculated for each NGC predicted subtype.

#### Layer-electrophysiological correlates

Overall correlation for each electrophysiological parameter on each NGC subtype was correlated (Pearson, R) with respect to the quantitative annotation of patch-seq cell radial position. To assess the relationship between each electrophysiological feature and each cortical layer (or sublayer) independently, χ^2^ Pearson residuals were calculated using the ‘chisq.test’ R function.

### Statistical analyses

All statistical analyses were performed using R standard libraries. Two-sided Kolmogorov–Smirnov test was used to assess statistical significance on histological quantifications aimed at interrogating differential cell population spatial distributions (Figs 1B, 5A, Fig. S9B). Unpaired *t*-test was used for comparing electrophysiological measurements between NGC subtypes. Hypergeometric tests were used for gene-set analysis to assess GO and HGNC gene family enrichments. Sample sizes were not pre-determined; statistical comparisons were conducted using the maximum sampling capacity at our disposal.

## Supplementary Material

10.1242/develop.201830_sup1 Supplementary information
