# Supplementary material for: Developmental emergence of cortical neurogliaform cell diversity
Source: Development. 2023 Aug 1;150(15):dev201830. doi: 10.1242/dev.201830 (PMC10445751; doi:10.1242/dev.201830)
Supplement: Supplementary information [file develop-150-201830-s1.pdf]

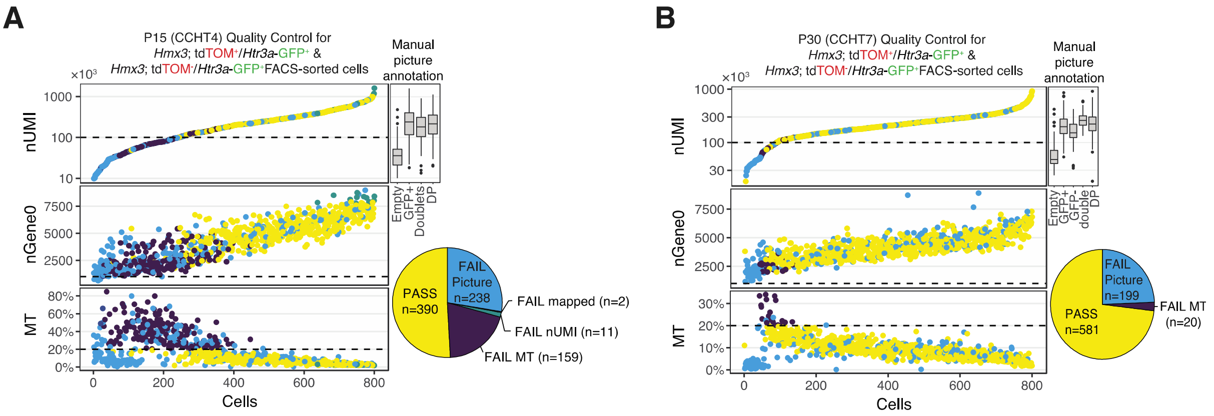

Fig. S1. Quality Control in postnatal scRNA-seq datasets. A-B. Scatter plots illustrating cell scores on Quality Control (QC) criteria applied to P15 and P30 microfluidic-based datasets containing both *Hmx3*-dtTOM$^+$; *Htr3a*-GFP$^+$ and *Hmx3*-dtTOM-; *Htr3a*-GFP$^+$ cells: number of UMIs detected (nUMI), number of genes detected (nGene0) and percentage of mitochondrial reads (MT) (color-coding detailed below). Boxplot illustrating nUMI distribution by microfluidic chamber type: empty, GFP$^+$ (*Hmx3*-dtTOM-;*Htr3a*-GFP$^+$), doublet or DP (*Hmx3*-dtTOM$^+$;*Htr3a*-GFP$^+$). Pie charts illustrating the proportion and number of cells by QC status (yellow: QC successfully passed (390 at P15, 581 at P30, blue, green or purple QC failed); blue: QC fail picture - bad quality picture or empty plate chamber; purple: QC fail - MT reads > 20%; green: QC fail - low nUMI count; light green: QC fail – low number of mapped reads).

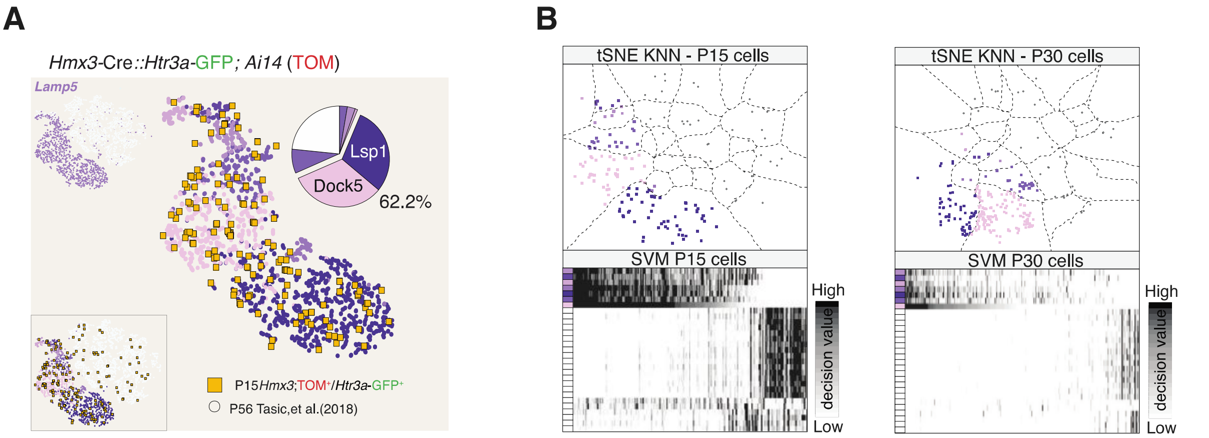

Fig. S2. Cell type assignment of UL NGCs and prediction confidence assessment. A. tSNE plot illustrating the integration of sequenced P15 NGCs that passed QC (*Hmx3*;tdTOM$^+$/*Htr3a*-GFP$^+$, n=196) onto a transcriptomic atlas of P56 cortical *Htr3a*-expressing INs (n=4743) (Tasic et al., 2018) (bottom tSNE inset covers all *Htr3a*-expressing IN subtypes, upper tSNE inset shows *Lamp5* expression among all Htr3a-expressing INs and main tSNE displays only *Lamp5*-expressing INs, n=1881). Pie chart displays the percentage of P15 NGCs mapping to the different *Htr3a*-expressing IN subtypes (highlighted NGCs fraction mapping to Dock5$^+$ NGC and Lsp1$^+$ NGC subtypes, 52.2% (n=36) and 47.8% (n=33) respectively; total 62.2% - 69 cells out of 111, remaining 85 cells failed to reach a consensus prediction). **B.** tSNE scatter plots and heatmaps depicting cell type prediction results for P15 and P30 *Hmx3*;tdTOM$^+$/*Htr3a*-GFP$^+$ cells: KNN assignment tSNE scatter plot with averaged decision frontiers trained on Tasic 2018 cells and SVM heatmap decision weights. Color-Shape-coding: Dock5$^+$NGCs (pink circles), Lsp1$^+$NGCs (purple circles), all *Hmx3*;tdTOM$^+$/*Htr3a*-GFP$^+$ cells (yellow squares), *Lamp5*$^+$ subtypes (shades of purple circles), *Lamp5*- subtypes (white circles), SVM decision values (gradient from white (low confidence) to black (high confidence)), *Lamp5* expression (gradient from white (lowest RPM) tp dark green (highest RPM)).

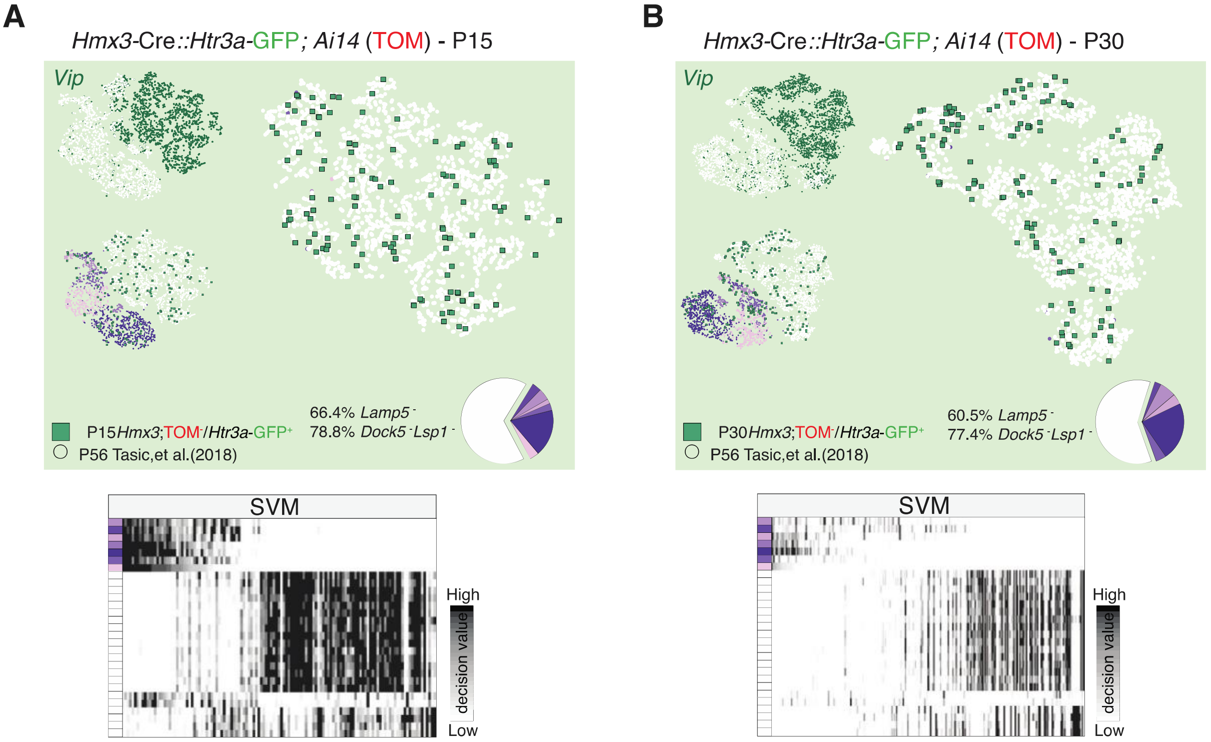

Fig. S3. Cell type assignment of non-NGC *Htr3a*-expressing INs. A. tSNE plot illustrating the integration of sequenced P15 non-NGC *Htr3a*$^+$ INs (*Hmx3*;tdTOM-/*Htr3a*-GFP$^+$ cells, n=194) onto a transcriptomic atlas of P56 cortical *Htr3a*-expressing INs (n=4743) (Tasic et al., 2018). Bottom tSNE inset covers mapping through all *Htr3a*-expressing IN subtypes, upper tSNE inset shows *Vip* expression and main tSNE displays only *Lamp5*$^-$ INs. Pie chart displaying the percentage of P15 non-NGCs mapping onto *Htr3a*-expressing IN subtypes (white highlighted pie portion shows non-NGCs mapping to *Lamp5*-negative subtypes, 66.4% - 75 out of 113 assigned cells, remaining 81 cells failed to reach a consensus prediction). *Dock5-Lsp1-* P15 non-NGCs constitute the 78.6% - 89 out of 113 assigned cells). Heatmap depicting cell type prediction results for P15 non-NGC cells: represented by SVM decision values (model weights). **B.** Similarly as in **A**, for P30 non-NGC *Htr3a*$^+$ INs (*Hmx3*;tdTOM-/*Htr3a*-GFP$^+$ cells, n=280) (map preferentially to *Lamp5*-negative subtypes, 60.5% - 75 out of 124 assigned cells (remaining 156 cells failed to reach a consensus prediction). *Dock5-Lsp1-* P30 non-NGCs constitute the 77.4% - 96 out of 124 cells). Color-Shape-coding: *Hmx3*;tdTOM-/*Htr3a*-GFP$^+$ cells (green squares), *Lamp5*$^+$ subtypes (shades of purple circles), *Lamp5-* subtypes (white circles), SVM decision values (gradient from white (low confidence) to black (high confidence)), *Vip* expression (gradient from white (lowest RPM) to dark green (highest RPM)).

Development • Supplementary information

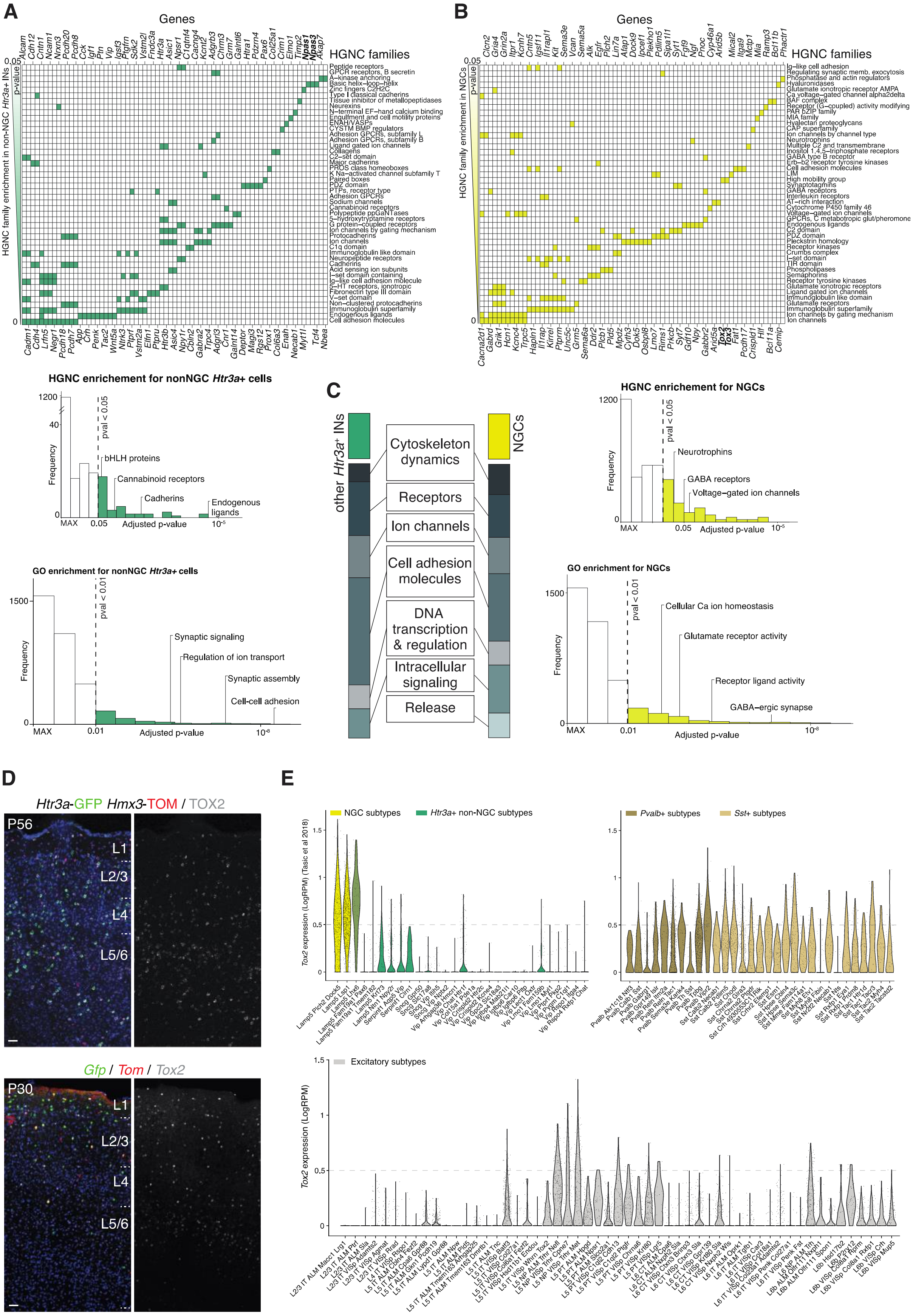

Fig. S4. Gene Enrichment Analysis on NGC vs other *Htr3a*+ INs time-conserved molecular signatures. A. Heatmap highlighting enriched HGNC families for SVM identified genes (columns) in

Development • Supplementary information

Htr3a+ INs other than NGCs (rows) (p-value filling, scale from white (low) to green (high)). Detailed information on gene enrichments here represented is available in Table S5. Histograms display p-value frequency bins for GO and HGNC items. Dashed vertical line signals significance threshold (lower than 0.05 for HGNC and lower than 0.01 for GO terms) and significant histogram bins are cell type-colored (yellow; white in histograms indicates no enrichment). **B.** Heatmap highlighting enriched HGNC families for SVM identified genes (columns) in NGCs (rows) (p-value filling, scale from white (low) to yellow (high)). Histograms display p-value frequency bins for GO and HGNC items. Dashed vertical line signals significance threshold (lower than 0.05 for HGNC and lower than 0.01 for GO terms) and significant histogram bins are cell type-colored (yellow; white in histograms indicates no enrichment). **C.** Bar-plot comparing proportion of enriched HGNC family groups for compared cell types' (NGCs in yellow versus other *Htr3a*$^{+}$ INs in green) (see Table S5 for a detailed view on HGNC family grouping). **D.** Example images of Tox2 mRNA expression at P56 (top) and TOX2 protein expression at P30 (bottom) in the cortex. **E.** Violin plots showing Tox2 mRNA expression in adulthood for all neuronal transcriptomic subtypes described in Tasic et al., 2018 (this third-party dataset and its nomenclature was used): NGCs (yellow), *Htr3a*-expressing non-NGCs INs (green), Pvalb-expressing INs (brown), Sst-expressing INs (beige), excitatory subtypes (grey); grey dashed line at 0.5 logRPM threshold. Scale bars: D, both, 50 μm.

Development • Supplementary information

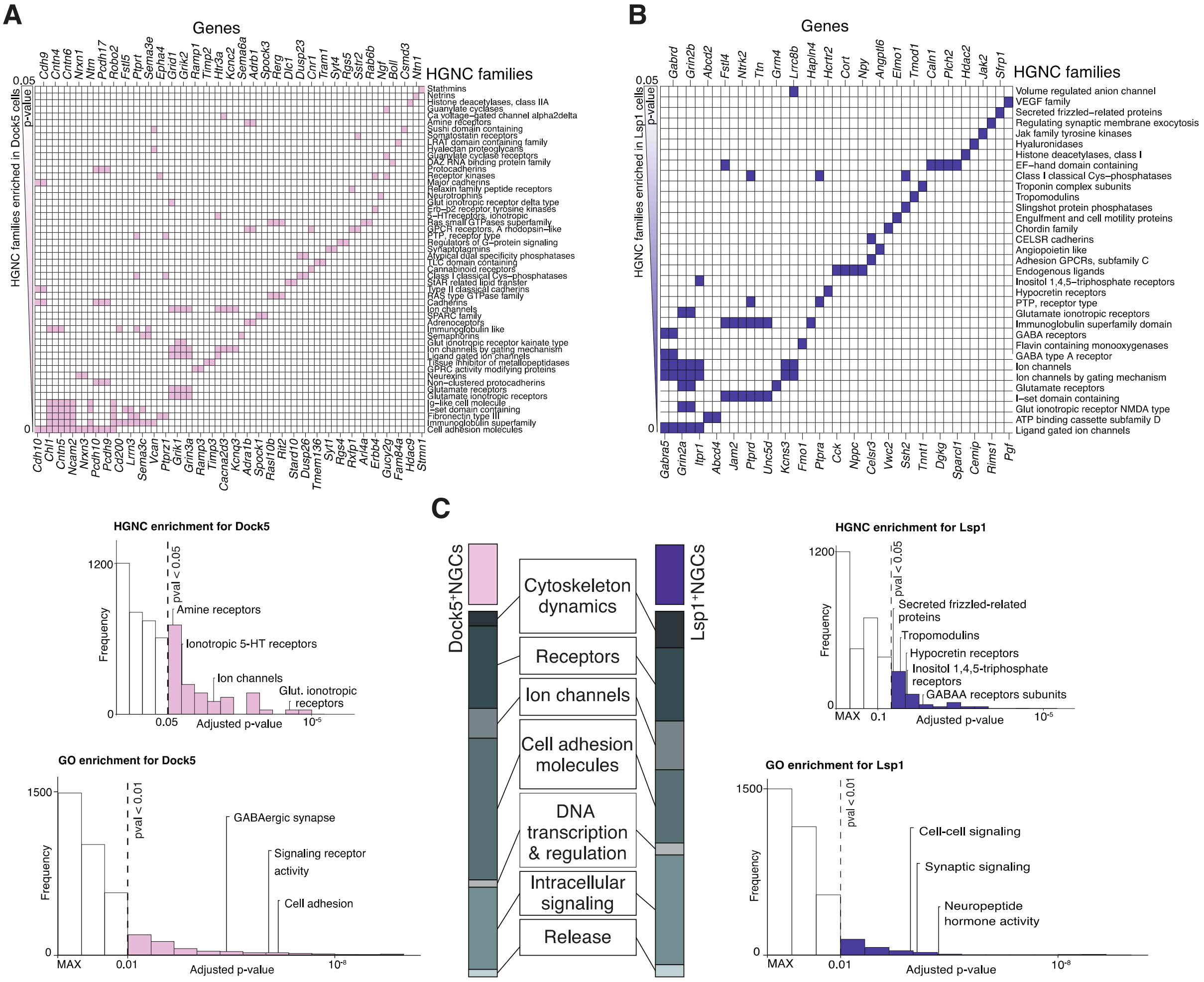

Fig. S5. Gene Enrichment Analysis on Dock5⁺NGCs vs Lsp1⁺NGCs time-conserved molecular signatures. A. Heatmap highlighting enriched HGNC families for SVM identified genes (columns) in Dock5⁺NGCs (rows) (p-value filling, scale from white (low) to pink (high)). Detailed information on gene enrichments here represented is available in Table S6. Histograms display p-value frequency bins for GO and HGNC items. Dashed vertical line signals significance threshold (lower than 0.05 for HGNC and lower than 0.01 for GO terms) and significant histogram bins are cell type-colored (pink; white in histograms indicates no enrichment). **B.** Heatmap highlighting enriched HGNC families for SVM identified genes (columns) in Lsp1⁺NGCs (rows) (p-value filling, scale from white (low) to purple (high)). Histograms display p-value frequency bins for GO and HGNC items. Dashed vertical line signals significance threshold (lower than 0.05 for HGNC and lower than 0.01 for GO terms) and significant histogram bins are cell type-colored (purple; white in histograms indicates no enrichment). **C.** Bar-plot comparing proportion of enriched HGNC family groups for compared cell types' (Dock5 ⁺NGCs in pink versus Lsp1⁺NGCs in purple) (see Table S6 for a detailed view on HGNC family grouping).

Development • Supplementary information

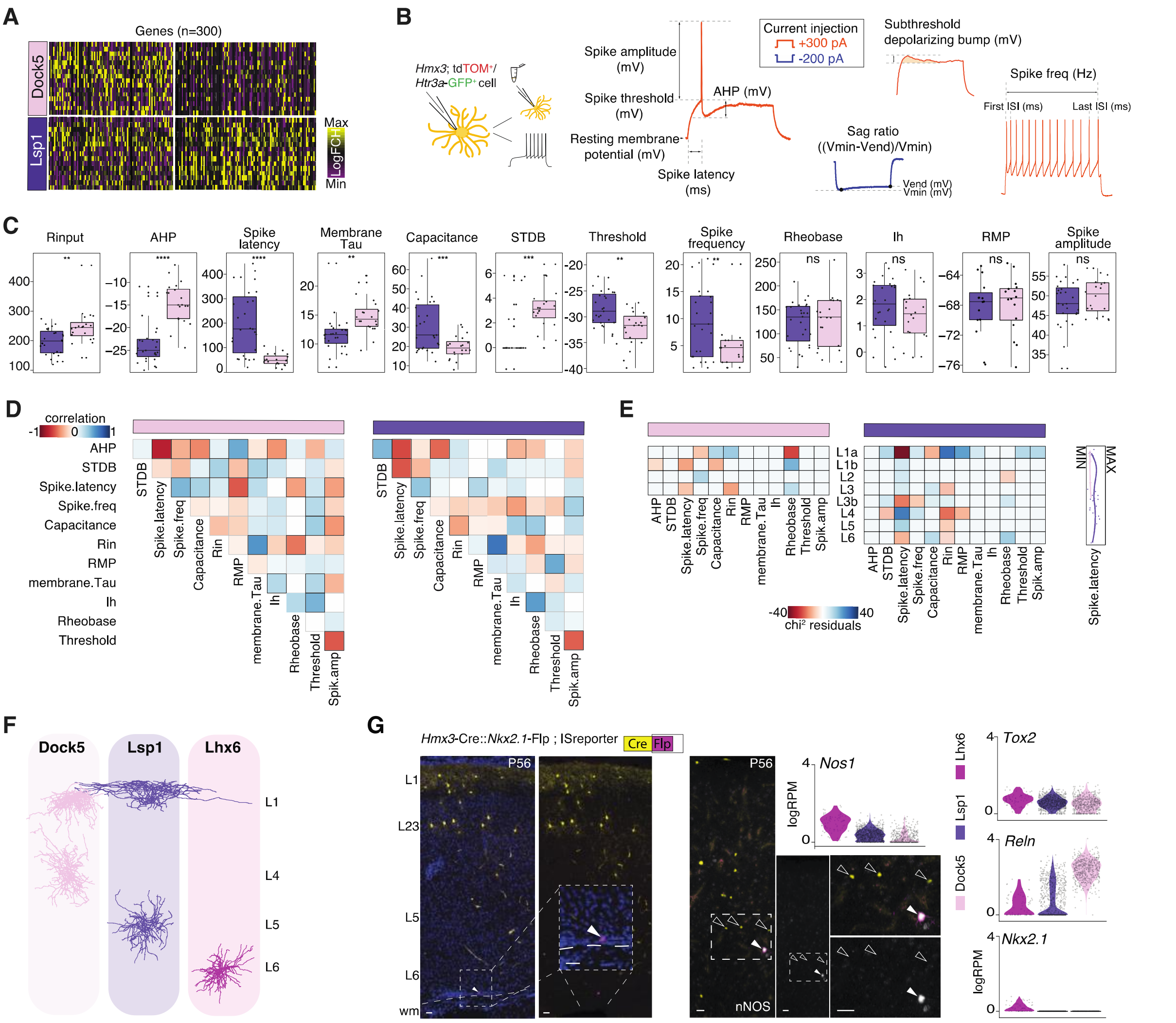

Fig. S6. Functional and anatomical correlates of NGC-subtypes. A. Heatmap illustrating the expression Fold Change for each patch-sequenced NGC and SVM gene (Dock5^{+}NGCs, pink; Lsp1^{+}NGCs, purple) (300 genes, 47 cells). **B.** Schematic representation of measured electrophysiological parameters in patch-seq protocol. **C.** Boxplots displaying the scores for each electrophysiological parameter for each single cell (split and color-coded by NGC subtype) (unpaired t-test; significance representation: ns: non-significative, *p<0.05, **p<0.01, ***p<0.001, ****p<0.0001; n=20 Dock5^{+}NGCs and n=27 Lsp1^{+}NGCs). **D.** Pearson correlation heatmap between the different electrophysiological features within each NGC subtype (correlation color-coding gradient: red (strong anticorrelation) - white (non-related) -blue (strong correlation)). **E.** Chi-square residuals heatmap illustrating the relation between the cortical layer of the patched NGC compared to electrophysiological parameters (residuals color-coding gradient: red (strong negative association) – white (non-associated) – blue (strong positive association)). Scatter-LOESS plot with NGC-subtype color-coding representing the cortical radial position of patched cells (y-axis) and their spike latency values (x-axis). **F.** Examples of morphological reconstructions for each NGC subtype across cortical layers, adapted from Scala et al., 2021 (published under a Creative Commons CC BY 4.0 license: https://creativecommons.org/licenses/by/4.0/). (color-coding represents NGC subtypes: Lhx6^{+}NGCs (dark pink), Lsp1^{+}NGCs (purple), Dock5^{+}NGCs (light pink). **G.** Example images of cortical section

Development • Supplementary information

displaying putative Lhx6+NGC subtype cells genetically fate-mapped using Hmx3-Cre::Nkx2.1-Flp; ISreporter mice and their nNOS protein expression. Violin plots representing logRPM expression values for genes of interest in Lhx6^{+}NGCs compared to other NGC subtypes (color-coding represents NGC subtypes: Lhx6^{+}NGCs (dark pink), Lsp1^{+}NGCs (purple), Dock5^{+}NGCs (light pink) (Tasic et al., 2018 labeled cells). Scale bars: G, low and high-magnification 25μm. Abbreviations: AHP (after hyperpolarization potential), STDB (subthreshold depolarizing bump), RMP (resting membrane potential), Rin (input resistance), Ih (hyperpolarization-activated cation current), amp (amplitude), freq (frequency), lat (latency), tau (membrane time constant), ISreporter (intersection-subtraction reporter).

Development • Supplementary information

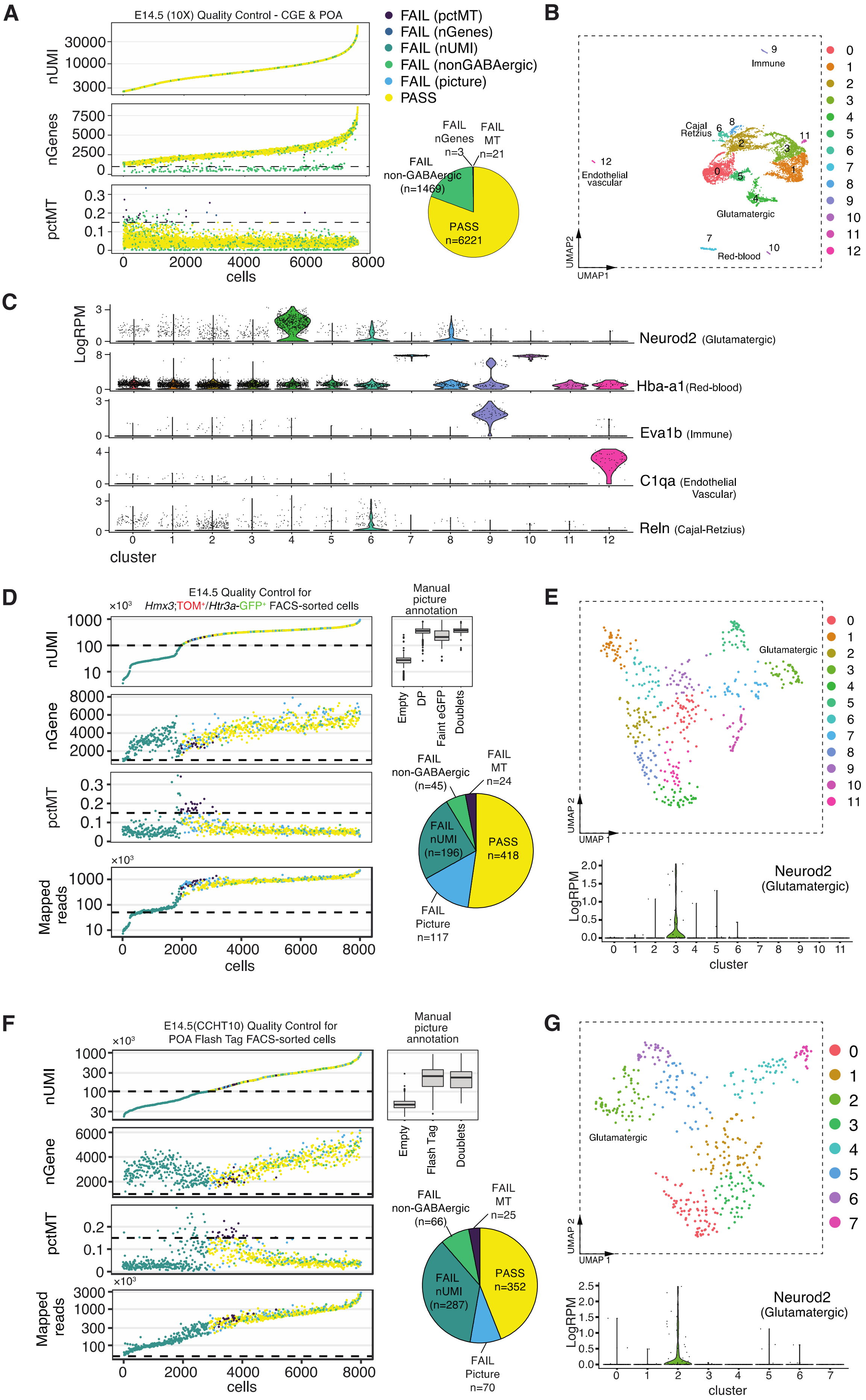

Development • Supplementary information

Fig. S7. Quality Control in embryonic scRNA-seq datasets. A. Scatter plots illustrating cell scores on Quality Control (QC) criteria applied to droplet-based datasets containing WT cells both from POA and CGE: number of UMIs detected (nUMI), number of genes detected (nGene0) and percentage of mitochondrial reads (MT). Pie charts illustrating the proportion and number of cells by QC status (yellow: QC successfully passed (4115 for CGE, 2106 for POA), QC failed - purple: QC fail - MT reads; green: QC fail - nUMI count; light green: QC fail – non-GABAergic). **B.** UMAP scatter plot illustrating cell types found among WT E14.5 CGE and POA datasets (text-labeled cell types were removed). **C.** LogRPM expression of illustrative marker on each UMAP cluster (B) removed for further analysis. **D.** Scatter plots illustrating cell scores on QC criteria applied to the FACS-sorted E14.5 dataset containing *Hmx3*;tdTOM^{+}/*Htr3a*-GFP^{+} cells. Boxplot illustrating nUMI distribution by microfluidic chamber type: empty, GFP^{+} (*Hmx3*-dtTOM-;*Htr3a*-GFP^{+}), doublet or DP (*Hmx3*-dtTOM^{+};*Htr3a*-GFP^{+}). Pie chart illustrating the proportion and number of cells by QC status (yellow: QC successfully passed (418), blue, green or purple QC failed); blue: QC fail picture - bad quality picture or empty plate chamber; purple: QC fail - MT reads; green: QC fail - nUMI count; light green: QC fail –non-GABAergic). **E.** UMAP scatter plot illustrating cell types found among *Hmx3*-dtTOM-;*Htr3a*-GFP^{+} E14.5 POA cells (text-labeled cell types were removed). LogRPM expression of illustrative marker on each UMAP cluster removed for further analysis. **F.** Scatter plots illustrating cell scores on QC criteria applied to the FACS-sorted E14.5 POA dataset containing 2+ Flash Tagged (FT) cells. Boxplot illustrating nUMI distribution by microfluidic chamber type: empty, FT^{+} or doublet. Pie chart illustrating the proportion and number of cells by QC status (yellow: QC successfully passed (352), blue, green or purple QC failed); blue: QC fail picture - bad quality picture or empty plate chamber; purple: QC fail - MT reads; green: QC fail - nUMI count; light green: QC fail –non-GABAergic). **G.** UMAP scatter plot illustrating cell types found among FT E14.5 POA cells (text-labeled cell types were removed). LogRPM expression of illustrative marker on each UMAP cluster removed for further analysis.

Development • Supplementary information

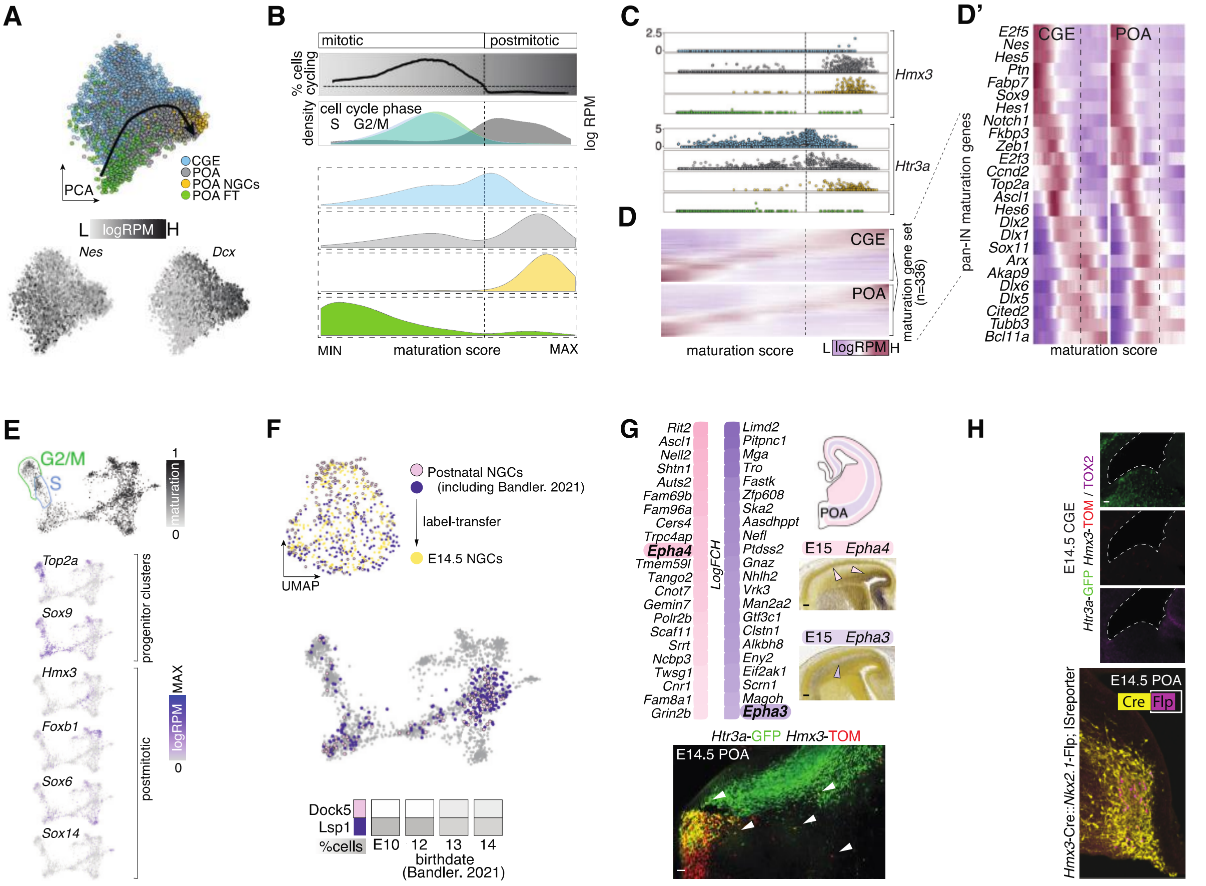

Fig. S8. NGC embryonic maturation and subtype characterization. **A.** PCA plots displaying cells from all collected E14.5 scRNA-seq datasets (CGE WT, n=4115, blue; POA WT, n=2106, grey; POA 2+ FT, n=352, green; POA *Hmx3*-dtTOM-;*Htr3a*-GFP$^+$, n=418, yellow) (Table S11, Table S12). Pseudotime fitted curve and expression of two time-genes: *Nes* and *Dcx,* early and late respectively (logRPM white (low) – grey (high) gradient). **B.** Scatter and density plots illustrating the reconstructed E14.5 maturation score (=pseudotime) according to the percentage of cycling cells and their cycle phase at each moment in development (S phase, blue; G2/M phases, green) (Table S11, Table S12). Jittered density plots showing the position in maturation of each cell color-coded and split by dataset (CGE, blue; POA WT, grey; POA FT, green; POA *Hmx3*-dtTOM-;*Htr3a*-GFP$^+$, yellow). **C.** Scatter plots showing the expression of genes used to fate-map NGCs across maturation score and datasets. **D.** Heatmap illustrating the set of genes used for reconstructing the POA&CGE maturation trajectory (n=336) (Table S13) ordered by their peak expression in pseudotime and color- coded by their logRPM value (violet (low) – white (mid) -brown (high) gradient). **D'** High-magnification of heatmap depicted in D highlighting the subset of genes described by Mayer et al., 2018 as maturation genes in MGE and LGE (pan-IN maturation genes). **E.** POA-dataset integration UMAP (Table S12) color-coded by: (top) maturation score (white (low) – black (high) gradient), and (bottom) cell cycle scores for S and G2M phases (black (low) – green (high) gradient), logRPM expression of cluster markers (grey (low) – blue (high) gradient). Markers belonging to progenitor or postmitotic clusters are detailed (Table S13). **F.** UMAP plot illustrating integration results for three datasets: Bandler, 2021 STICR, postnatal NGCs and embryonic NGCs (Table S13). Shape/color-coding: pink circles, Bandler Dock5$^+$NGCs; purple circles, Bandler Lsp1$^+$NGCs; pink squares, postnatal Dock5$^+$NGCs; purple squares, postnatal Lsp1$^+$NGCs;

Development • Supplementary information

yellow circles, embryonic NGCs. POA UMAP highlighting E14.5 cells belonging to the NGC lineage color-coded by subtype label-transfer. Heatmap illustrating the percentage of Bandler, 2021 cells by NGC subtype and birthdate (color-filling white (low) – grey (high) gradient) (% subtype birthdate - E10: 100% $Lsp1^{+}$NGCs, 4% of total; E12: 100% $Lsp1^{+}$NGCs, 5% of total; E13: 65% $Lsp1^{+}$NGCs, 30% of total; E14: 66% $Lsp1^{+}$NGCs, 30% of total). **G.** Heatmap representing gene-expression fold change for the most differentially expressed genes between postmitotic NGC subtypes at E14.5 (color-filling gradient of pink for $Dock5^{+}$NGCs and gradient of purple for $Lsp1^{+}$NGCs). Gene names are color-coded by their predicted localization in the nucleus (red) or cytoskeleton/projection (blue). Schematic of putative migratory routes used by cells expressing Eph receptors A4 and A3. Allen Brain Atlas ISH pictures of sagittal E15 and E18 brain sections stained against *Epha4* and *Epha3* mRNAs. High magnification and low magnification pictures of E14.5 coronal brain section from the *Htr3a*-GFP; *Hmx3*-tdTOM mouse line highlighting putative NGCs (white arrowheads). **H.** *Htr3a*-GFP; *Hmx3*-tdTOM E14.5 coronal section illustrating TOX2 protein absence among CGE cells (magenta). POA E14.5 coronal section from the *Hmx3*-Cre;*Nkx2.1*-Flp;ISreporter intersectional mouse line indicating the microdomain of the mixed *Hmx3* and *Nkx2.1* lineage (magenta Hmx3 $^{+}$Nkx2.1^{+} cells; yellow, $Hmx3^{+}$Nkx2.1- cells). Scale bars: G, Low magnification: 100μm, High magnification: 25μm; H, High and Low magnification: 25μm.

Development • Supplementary information

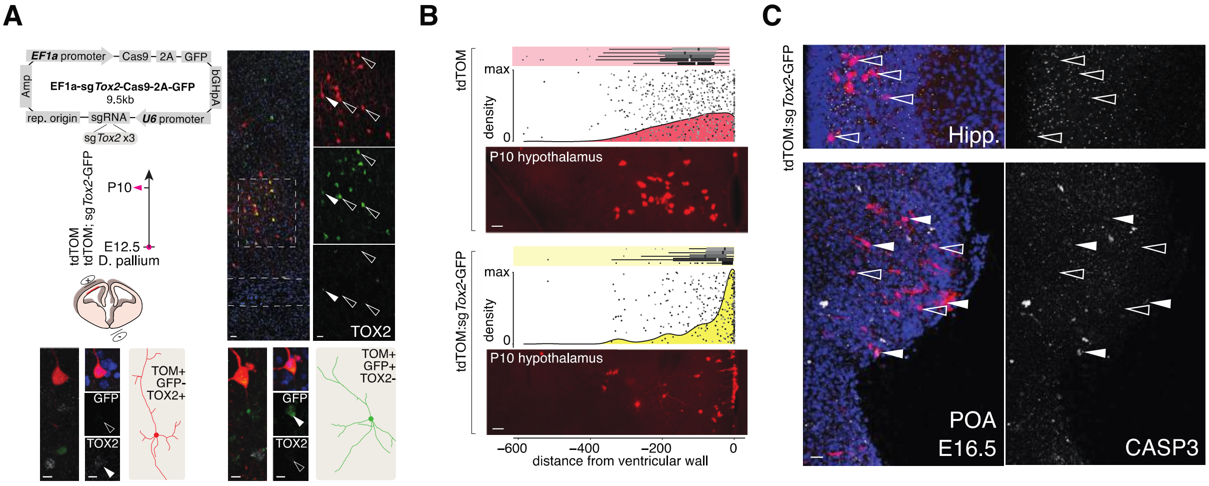

Fig. S9. Characterization of in-utero POA electroporation. A. Schematic illustrating the design of the sg*Tox2*-GFP plasmid containing 3 single guidance sequences. CRISPR strategy validation: schema illustrating *in-utero electroporation* experimental strategy and analysis pipeline for downregulating *Tox2* in dorsal pallium at E12.5. High- and Low-magnification pictures of P10 cortex. E12.5-electroporated excitatory neurons in L5/6 ($GFP^{+}TOM^{+}$, yellow in merged) are lacking TOX2 protein expression (empty arrowheads). High-magnification pictures of L5 example electroporated pyramidal cells and their morphological reconstruction. Left: tdTOM control cell; right: TOM^{+};sg*Tox2*-GFP^{+} CRISPR-targeted cell. **B.** Microscopy pictures of hypothalamus at P10 after E14.5 POA *in-utero* electroporation. Density/scatter plots illustrating position of electroporated cells quantified (cell distance in pixels from the ventricular surface) (Table S15). Biological replicates split in inset boxplots (n=5 brains per condition, 620 tdTOM control, 372 sg*Tox2* cells). **C.** Pictures of CASP3 protein expression in embryonic hippocampus and POA at E16.5, following tdTOM;sg*Tox2*GFP *in-utero* electroporation. Arrowheads indicate electroporated cells $CASP3^{+}$; empty arrowheads cells CASP3-. Scale bars: A, top panel - 25µm, bottom panel - 15µm; B, C 25µm.

Table S1. Postnatal scRNAseq QC, metadata and cell type assignment.

Click here to download Table S1

Table S2. Postnatal scRNAseq cell type enrichment.

Click here to download Table S2

Table S3. Quantification of smFISH against *Dock5* and *Lsp1* in fate-mapped P56 cortical NGCs.

Click here to download Table S3

Table S4. Core Postnatal cells maturation score and labeling.

Click here to download Table S4

Table S5. *Htr3a*$^{+}$IN Type (NGC vs other *Htr3a*$^{+}$ INs) molecular architectures, GO and HGNC gene enrichment analysis.

Click here to download Table S5

Table S6. NGC Subtype (Dock5^{+}NGCs vs Lsp1^{+}NGCs) molecular architectures, GO and HGNC gene enrichment analysis.

Click here to download Table S6

Table S7. *Tox2* smFISH quantification in fate-mapped P56 postnatal cortical NGCs.

Click here to download Table S7

Table S8. *Rxfp1* smFISH quantification in fate-mapped P56 postnatal cortical NGCs.

Click here to download Table S8

Table S9. Postnatal patch-seq QC, metadata and cell type assignment.

Click here to download Table S9

Table S10. Postnatal patch-seq intrinsic properties and PCA coordinates, subtype statistics, electrophysiology-layer correlations, gene-electrophysiology correlations and functional network analysis.

Click here to download Table S10

Table S11. E14.5 CGE scRNAseq QC statistics, metadata, pseudotime and PCA coordinates.

Click here to download Table S11

Table S12. E14.5 POA scRNAseq QC statistics, metadata, pseudotime, PCA and UMAP coordinates, cluster assignment and NGC type assignment.

Click here to download Table S12

Table S13. Pseudotime genes, POA cluster fate-mapping enrichment, cluster TF markers, NGC pseudogene Zscore across development, lineage cell type assignment, postmitotic markers NGC subtypes.

Click here to download Table S13

Table S14. Tox2 IHC quantification in fate-mapped E14.5 NGCs.

Click here to download Table S14

Table S15. *In-utero*-electroporation quantifications and analyisis.

Click here to download Table S15

Table S16. List of Primary Antibodies and RNA-Scope probes.

Click here to download Table S16
