# Supplementary material for: Developmental emergence of cortical neurogliaform cell diversity
Source: Development. 2023 Aug 1;150(15):dev201830. doi: 10.1242/dev.201830 (PMC10445751; doi:10.1242/dev.201830)
Supplement: Supplementary file 1 [file develop-150-201830_review_history.pdf]

Developmental emergence of cortical neurogliaform cell diversity

Lucia Gomez, Christelle Cadilhac, Julien Prados, Nandkishor Mule, Denis Jabaudon and Alexandre Dayer
DOI: 10.1242/dev.201830

Editor: Debra Silver

Review timeline

Original submission:	5 April 2023
Editorial decision:	19 May 2023
First revision received:	15 June 2023
Accepted:	17 June 2023

Original submission

First decision letter

MS ID#: DEVELOP/2023/201830

MS TITLE: Developmental emergence of cortical neurogliaform cell diversity

AUTHORS: Lucia Gomez, Christelle Cadilhac, Julien Prados, Nandkishor Kisanrao Mule, Alexandre Dayer, and Denis Jabaudon

I have now received all the referees reports on the above manuscript, and have reached a decision. The referees' comments are appended below, or you can access them online: please go to BenchPress and click on the 'Manuscripts with Decisions' queue in the Author Area.

The overall evaluation is positive and we would like to publish a revised manuscript in Development, provided that the referees' comments can be satisfactorily addressed. In particular, both reviewers had valuable suggestions for clarifications to the text. Reviewer 2 also suggested that for several experiments, the authors clarify proportions of labeled cells. Please attend to all of the reviewers' comments in your revised manuscript and detail them in your point-by-point response. If you do not agree with any of their criticisms or suggestions explain clearly why this is so. If it would be helpful, you are welcome to contact us to discuss your revision in greater detail. Please send us a point-by-point response indicating your plans for addressing the referee's comments, and we will look over this and provide further guidance.

Reviewer 1

Advance summary and potential significance to field

This is an interesting manuscript describing the molecular and physiological heterogeneity of neurogliaform cells (NGCs) and their emergence during embryogenesis in the mouse. The authors purified single cells from P15 and P30 brains from transgenic mice that label pre-optic area (POA)-derived NGCs and through mapping onto adult populations, were able to segregate them onto two transcriptional subtypes: one that expresses Dock5 and one that expresses Lsp1. The two have distinct distributions in L1 and lower layers (L2/3 and lower) respectively. They also have distinct molecular signatures and distinct electrophysiological properties. Purification and single cell RNA sequencing of progenitors and postmitotic cells from the embryonic POA showed that these molecular signatures emerge gradually. One of the markers that the authors have identified in

© 2023. Published by The Company of Biologists under the terms of the Creative Commons Attribution License (https://creativecommons.org/licenses/by/4.0/).

NGCs is Tox2. This transcription factor is expressed from the early stages of their development, through to adult. Using CRISPR technology and in utero electroporation the authors knocked out Tox2 at E14.4 in mouse embryos and showed loss of Tox2 expression, cell death and loss of electroporated cells indicating that Tox2 is required from the early stages for proper development of POA cells.

The manuscript is very data-rich with excellent illustrations throughout. It constitutes a great new addition to the NGC diversity and their embryonic emergence literature. The only drawback is the rather inadequate characterization of the role of Tox2 in NGC development. However, given the novelty of the finding that Tox2 labels NGCs, it is interesting to report.

Comments for the author

I only have a few suggestions:
1. Is Tox2 expressed only in NGCs? Presumably not, since the authors briefly mention validation of the KO strategy in pyramidal cells. For clarification, this point should be made clearer to avoid a misleading impression that Tox2 is specific to NGCs. If possible, supplementary images showing expression elsewhere in the brain should be included.

2. Figures S9B and S9C should be transferred to the main figure 5. It is important to show in the main manuscript the cell death and the changes in the POA following Tox2 KO.

3. In figure 5A, the plot illustrating the number of electroporated cells found in POA at E16.5 should be removed given that the sample size is small and the variation is large. The data can just be included in the text. Also, in the same figure, is the y axis in the Density scatter plots the same for control and sgTox2?

3. Some figure legends are using different fonts. This should be corrected.

Reviewer 2

Advance summary and potential significance to field

The manuscript entitled “Developmental emergence of cortical neurogliaform cell diversity” by Gomez and Cadilhac et al. examines the specification and maturation of cortical neurogliaform cells (NGFC), a population of GABAergic interneurons (INs) that have distinct developmental origins from other IN subtypes (marked by PV, SST or VIP) in that they emerge from both Nkx2.1+ (“MGE”) and non-Nkx2.1+ (“CGE/POA”) lineages in the cortex and hippocampus. Using a transgenic labeling strategy this group has published previously (e.g., Niquille et al., 2018), along with transcriptomic and electrophysiological analyses, the authors provide novel and interesting insights into NGFC diversity in the neocortex. Furthermore, they identify the transcription factor Tox2 as being expressed in NGFC embryonic precursors, and show that it is required for NGFC development. This study is a thorough and impactful addition to this group’s previous work, and honors Dr. Dayer’s scientific legacy.

Comments for the author

Specific comments:
1: This study relies extensively on BAC transgenic lines, in particular Hmx3(BAC)-Cre and Htr3a(BAC)-EGFP. It is important to note that while these types of genetic tools can be extremely useful to delineate populations of cells, they often do not accurately recapitulate the expression of the endogenous gene. For example, while the Htr3a(BAC)-EGFP expresses GFP in NGFC, the latter do not actually express significant levels of Htr3a in adult animals, as confirmed by the authors transcriptomic analysis here (Fig. 3B). Thus it is important to note this early on in the text, e.g., Intro lines 90-91 should be “Hmx3(BAC)-Cre::Htr3a(BAC)-EGFP; Ai14” to avoid confusion.

© 2023. Published by The Company of Biologists under the terms of the Creative Commons Attribution License (https://creativecommons.org/licenses/by/4.0/).

2: The authors combine Hmx3(BAC)-Cre; Ai14 (tdTomato reporter) with Htr3a(BAC)-EGFP to label "POA-born" cells. They isolate these cells, perform scRNAseq, and obtain 301 cells after QC, which they then assign to the subtypes defined in Tasic et al., 2018. Over half (154 cells) could not be assigned; the remaining cells (147) were comprised of Lamp5/Dock5 (76 cells; 51.7%), Lamp5/Lsp1 (35 cells; 23.8%), and other subtypes (36 cells; 24.5%). It would be useful to know the fraction of the total Lamp5/Dock5 and Lamp5/Lsp1 populations that the Hmx(BAC)-Cre; Ai14 cross is labeling, i.e., how efficient is this genetic approach in labeling NGFC?

3: The laminar distributions of Lamp5/Dock5 and Lamp5/Lsp1 populations (Fig. 1B) have been described previously in Tasic et al., 2018. The authors should mention that the Lamp5/Dock5 population roughly corresponds to NDNF+ NGFC, mostly located in L1, whereas Lamp5/Lsp1 correspond to non-NDNF NGFC mostly located in L2-6. The statement that NDNF and NPY are "classically considered as being expressed in all NGFC" (lines 147-8) is not accurate and not supported by the citations there; while relatively high levels of NPY expression does appear to be a common property of NGFC across all layers, NDNF expression is mostly restricted to L1 NGFC (e.g., Tasic et al., 2016). It would be helpful to know what fraction of L1 interneurons are labeled with Hmx(BAC)-Cre; Ai14, and whether canopy cells (non-NGFC NDNF cells in L1 that express low levels of NPY; Schuman et al., 2019) are also labeled with this approach.
Perhaps the authors already have an estimate of the percentage of Lamp5 cells in L1 that are labeled in the Hmx(BAC)-Cre; Ai14 cross?

4: The transcriptomic comparison of Hmx(BAC)-Cre; Ai14; Htr3a(BAC)-EGFP (mostly NGFC) with the non-labeled Htr3a(BAC)-EGFP cells (mostly VIP types) is very interesting, but the authors should be careful with their interpretation of the functional significance of differentially expressed genes. For example, Syt1 Syt7 and Rims1 (line 144) are widely expressed in other interneurons (e.g., see celltypes.brain-map.org/rnaseq), including PV and SST subtypes, so are probably not "specialized NGC molecular machineries for GABA volumetric release" (line 145).

5: The patch-seq work is very impressive but in comparing Dock5 vs. Lsp1 populations, the authors should take note of the laminar locations of the recorded cells. Schuman et al. recorded in L1 specifically, and made a distinction between NGFC and canopy cell types, the latter type being almost exclusively restricted to L1. The Lamp5/Dock5 population overall is therefore probably a mix of NDNF NGFC and canopy cells. It would be interesting to compare the Lamp5/Dock5 and Lamp5/Lsp1 cells that were recorded specifically in L2/3 to avoid the complexities of L1 and gain more insight into NGFC heterogeneity.

6: The identification and loss-of-function (LOF) experiments with Tox2 are very interesting, and provide novel insights into NGFC development. Does the Tox2 LOF approach preserve Tox3 expression? Perhaps the authors can speculate on whether the different Tox proteins (Tox, Tox2, Tox3) perform distinct or redundant roles in interneuron subtype specification. It should be noted that the Tox proteins are also expressed in subsets of other MGE derived interneurons (celltypes.brain-map.org/rnaseq) and thus may function in several contexts to guide cell type diversity.

First revision

Author response to reviewers' comments

Reviewer 1
This is an interesting manuscript describing the molecular and physiological heterogeneity of neurogliaform cells (NGCs) and their emergence during embryogenesis in the mouse. The authors purified single cells from P15 and P30 brains from transgenic mice that label pre- optic area (POA)-derived NGCs and, through mapping onto adult populations, were able to segregate them onto two transcriptional subtypes: one that expresses Dock5 and one that expresses Lsp1. The two have distinct distributions in L1 and lower layers (L2/3 and lower) respectively. They also have distinct molecular signatures and distinct electrophysiological properties. Purification and single cell RNA sequencing of progenitors and postmitotic cells from the embryonic POA showed that these molecular signatures emerge gradually. One of the markers that the authors have identified in

© 2023. Published by The Company of Biologists under the terms of the Creative Commons Attribution License (https://creativecommons.org/licenses/by/4.0/).

NGCs is Tox2. This transcription factor is expressed from the early stages of their development, through to adult. Using CRISPR technology and in utero electroporation the authors knocked out Tox2 at E14.4 in mouse embryos and showed loss of Tox2 expression, cell death and loss of electroporated cells, indicating that Tox2 is required from the early stages for proper development of POA cells.

The manuscript is very data-rich with excellent illustrations throughout. It constitutes a great new addition to the NGC diversity and their embryonic emergence literature. The only drawback is the rather inadequate characterization of the role of Tox2 in NGC development. However, given the novelty of the finding that Tox2 labels NGCs, it is interesting to report.

We thank the Reviewer for their comments and suggestions to improve our manuscript.

Reviewer 1 Comments for the Author:
I only have a few suggestions:

1. Is Tox2 expressed only in NGCs? Presumably not, since the authors briefly mention validation of the KO strategy in pyramidal cells. For clarification, this point should be made clearer to avoid a misleading impression that Tox2 is specific to NGCs. If possible, supplementary images showing expression elsewhere in the brain should be included.

Tox2 is indeed expressed in other cell types. We have now made this point clearer in the text (lines 150-152), and have added two panels in the Supplementary Fig. S4 to illustrate this expression.

2. Figures S9B and S9C should be transferred to the main figure 5. It is important to show in the main manuscript the cell death and the changes in the POA following Tox2 KO.

We thank the reviewer for this suggestion. We feel, however, that this might be confusing to the reader as Fig. 5 is focused on NGCs while Figs. S9B and S9C illustrate the situation for other cell types. Given that the topic of cell death and global changes in the POA is well covered in the main text (each of these panels is referred to by a dedicated sentence), we would rather keep the text as such.

3. In figure 5A, the plot illustrating the number of electroporated cells found in POA at E16.5 should be removed given that the sample size is small and the variation is large. The data can just be included in the text. Also, in the same figure, is the y axis in the Density scatter plots the same for control and sgTox2?

Panel 5A has been removed and the data have now been introduced in the main text. The scale of the density scatter Y axis has been clarified in the legend (identical for both plots).

4. Some figure legends are using different fonts. This should be corrected.

We went through the figures and made sure items and fonts were displayed properly.

Reviewer 2
The manuscript entitled “Developmental emergence of cortical neurogliaform cell diversity” by Gomez and Cadilhac et al. examines the specification and maturation of cortical neurogliaform cells (NGFC), a population of GABAergic interneurons (INs) that have distinct developmental origins from other IN subtypes (marked by PV, SST or VIP) in that they emerge from both Nkx2.1+ (“MGE”) and non-Nkx2.1+ (“CGE/POA”) lineages in the cortex and hippocampus. Using a transgenic labeling strategy this group has published previously (e.g., Niquille et al., 2018), along with transcriptomic and electrophysiological analyses, the authors provide novel and interesting insights into NGFC diversity in the neocortex. Furthermore, they identify the transcription factor Tox2 as being expressed in NGFC embryonic precursors, and show that it is required for NGFC development. This study is a thorough and impactful addition to this group’s previous work, and honors Dr. Dayer’s scientific legacy.

© 2023. Published by The Company of Biologists under the terms of the Creative Commons Attribution License (https://creativecommons.org/licenses/by/4.0/).

We thank the Reviewer for their comments and suggestions to improve our manuscript.

Reviewer 2 Comments for the Author:
Specific comments:

1: This study relies extensively on BAC transgenic lines, in particular Hmx3(BAC)-Cre and Htr3a(BAC)- EGFP. It is important to note that while these types of genetic tools can be extremely useful to delineate populations of cells, they often do not accurately recapitulate the expression of the endogenous gene. For example, while the Htr3a(BAC)-EGFP expresses GFP in NGFC, the latter do not actually express significant levels of Htr3a in adult animals, as confirmed by the authors transcriptomic analysis here (Fig. 3B). Thus it is important to note this early on in the text, e.g., Intro lines 90-91 should be "Hmx3(BAC)- Cre::Htr3a(BAC)-EGFP; Ai14" to avoid confusion.

We have corrected our nomenclature and have added a sentence regarding potential limitations of this transgenic line (lines 375-392). We would like to clarify that Fig 3B does not illustrate *Htr3a* expression levels but correlation values between gene expression and electrophysiological features. This has now been made clearer in the text.

2: The authors combine Hmx3(BAC)-Cre; Ai14 (tdTomato reporter) with Htr3a(BAC)- EGFP to label "POA- born" cells. They isolate these cells, perform scRNAseq, and obtain 301 cells after QC, which they then assign to the subtypes defined in Tasic et al., 2018. Over half (154 cells) could not be assigned; the remaining cells (147) were comprised of Lamp5/Dock5 (76 cells; 51.7%), Lamp5/Lsp1 (35 cells; 23.8%), and other subtypes (36 cells; 24.5%). It would be useful to know the fraction of the total Lamp5/Dock5 and Lamp5/Lsp1 populations that the Hmx(BAC)-Cre; Ai14 cross is labeling, i.e., how efficient is this genetic approach in labeling NGFC?

The fraction of total labelled cells labelled is now provided in the text (lines 121-124). Amongst all $Lamp5^{+}Dock5^{+}$ and $Lamp5^{+}Lsp1^{+}$ cells assigned with both mouse lines, the vast majority were labeled by the *Hmx3*-Cre;tdTOM approach, suggesting a high efficiency of labeling for these subtypes (at P30: 79.8%; 100% $Lamp5^{+}Dock5^{+}$ and 55.5% $Lamp5^{+}Lsp1^{+}$; at P15: 74.2%; 90% $Lamp5^{+}Dock5^{+}$ and 62.3% $Lamp5^{+}Lsp1^{+}$).

3: The laminar distributions of Lamp5/Dock5 and Lamp5/Lsp1 populations (Fig. 1B) have been described previously in Tasic et al., 2018. The authors should mention that the Lamp5/Dock5 population roughly corresponds to NDNF+ NGFC, mostly located in L1, whereas Lamp5/Lsp1 correspond to non-NDNF NGFC mostly located in L2-6. The statement that NDNF and NPY are "classically considered as being expressed in all NGFC" (lines 147-8) is not accurate and not supported by the citations there; while relatively high levels of NPY expression does appear to be a common property of NGFC across all layers, NDNF expression is mostly restricted to L1 NGFC (e.g., Tasic et al., 2016). It would be helpful to know what fraction of L1 interneurons are labeled with Hmx(BAC)-Cre; Ai14, and whether canopy cells (non-NGFC NDNF cells in L1 that express low levels of NPY; Schuman et al., 2019) are also labeled with this approach.
Perhaps the authors already have an estimate of the percentage of Lamp5 cells in L1 that are labeled in the Hmx(BAC)-Cre; Ai14 cross?

We now mention in the text that the Lamp5/Dock5 population roughly corresponds to NDNF+ NGFC, mostly located in L1, whereas Lamp5/Lsp1 correspond to non-NDNF NGFC mostly located in L2-6 (lines 161-164).
The fraction of L1 interneurons labelled with Hmx(BAC)-Cre; Ai14 is reported in Niquille et al., 2018 Figure 2 and corresponding source data, and is 35% at P21 (virtually all L1 INs are *Htr3a*-expressing Tremblay et al., 2016). This is now mentioned in the text (line 303).
Based on the presence of LAMP5+ DOCK5+ low NPY cells in the transcriptomic data (see Fig. 2B), canopy cells are also labelled with this approach. This is now mentioned in the text (lines 161-164).
We cannot determine the actual percentage of labeled Lamp5 cells because our experimental

© 2023. Published by The Company of Biologists under the terms of the Creative Commons Attribution License (https://creativecommons.org/licenses/by/4.0/).

setup for scRNA-seq has been designed to enrich for “minor” populations of cells, i.e. NGCs, such that ratios of other cell types will be biased.

4: The transcriptomic comparison of Hmx(BAC)-Cre; Ai14; Htr3a(BAC)-EGFP (mostly NGFC) with the non- labeled Htr3a(BAC)-EGFP cells (mostly VIP types) is very interesting, but the authors should be careful with their interpretation of the functional significance of differentially expressed genes. For example, Syt1, Syt7 and Rims1 (line 144) are widely expressed in other interneurons (e.g., see celltypes.brain- map.org/rnaseq), including PV and SST subtypes, so are probably not “specialized NGC molecular machineries for GABA volumetric release” (line 145).

We have now modified this sentence to :
“ potentially reflecting specificities in ~~NGCs refined usage of specialized NGC molecular machineries for GABA neurotransmitter volumetric~~ vesiclee xocyto sis”

5: The patch-seq work is very impressive but in comparing Dock5 vs. Lsp1 populations, the authors should take note of the laminar locations of the recorded cells. Schuman et al. recorded in L1 specifically, and made a distinction between NGFC and canopy cell types, the latter type being almost exclusively restricted to L1. The Lamp5/Dock5 population overall is therefore probably a mix of NDNF NGFC and canopy cells. It would be interesting to compare the Lamp5/Dock5 and Lamp5/Lsp1 cells that were recorded specifically in L2/3 to avoid the complexities of L1 and gain more insight into NGFC heterogeneity.

The lamina specific properties of recorded cells (provided in Fig S6E and discussed in main text) revealed some level of phenotypic variation with laminar depth. We added a clarification in the text with reference to canopy cells (line 208).

6: The identification and loss-of-function (LOF) experiments with Tox2 are very interesting, and provide novel insights into NGFC development. Does the Tox2 LOF approach preserve Tox3 expression? Perhaps the authors can speculate on whether the different Tox proteins (Tox, Tox2, Tox3) perform distinct or redundant roles in interneuron subtype specification. It should be noted that the Tox proteins are also expressed in subsets of other MGE derived interneurons (celltypes.brain- map.org/rnaseq) and thus may function in several contexts to guide cell type diversity.

We have no information on Tox3 expression following LOF of Tox2. There is no reported cross-regulation between these and their function in interneuron subtype specification. However, evidence in immunology research suggests that Tox and Tox2 cooperate upregulating PD-1 expression (Seo, H. et al., 2019). We have added a sentence on the existence of other Tox family members and their potential functions (lines 350-351 and 360-365).

Second decision letter

MS ID#: DEVELOP/2023/201830

MS TITLE: Developmental emergence of cortical neurogliaform cell diversity

AUTHORS: Lucia Gomez, Christelle Cadilhac, Julien Prados, Nandkishor Kisanrao Mule, Alexandre Dayer, and Denis Jabaudon
ARTICLE TYPE: Research Article

I am happy to tell you that your manuscript has been accepted for publication in Development, pending our standard ethics checks.

© 2023. Published by The Company of Biologists under the terms of the Creative Commons Attribution License (https://creativecommons.org/licenses/by/4.0/).
